# DHA Improves Cognition and Prevents Dysfunction of Entorhinal Cortex Neurons in 3xTg-AD Mice

**DOI:** 10.1371/journal.pone.0017397

**Published:** 2011-02-23

**Authors:** Dany Arsenault, Carl Julien, Cyntia Tremblay, Frédéric Calon

**Affiliations:** 1 Faculté de pharmacie, Université Laval, Québec, Québec, Canada; 2 Centre de Recherche du CHUL (CHUQ) Québec, Québec, Québec, Canada; Biological Research Center of the Hungarian Academy of Sciences, Hungary

## Abstract

Defects in neuronal activity of the entorhinal cortex (EC) are suspected to underlie the symptoms of Alzheimer's disease (AD). Whereas neuroprotective effects of docosahexaenoic acid (DHA) have been described, the effects of DHA on the physiology of EC neurons remain unexplored in animal models of AD. Here, we show that DHA consumption improved object recognition (↑12%), preventing deficits observed in old 3xTg-AD mice (↓12%). Moreover, 3xTg-AD mice displayed seizure-like akinetic episodes, not detected in NonTg littermates and partly prevented by DHA (↓50%). Patch-clamp recording revealed that 3xTg-AD EC neurons displayed (i) loss of cell capacitance (CC), suggesting reduced membrane surface area; (ii) increase of firing rate versus injected current (F-I) curve associated with modified action potentials, and (iii) overactivation of glutamatergic synapses, without changes in synaptophysin levels. DHA consumption increased CC (↑12%) and decreased F-I slopes (↓21%), thereby preventing the opposite alterations observed in 3xTg-AD mice. Our results indicate that cognitive performance and basic physiology of EC neurons depend on DHA intake in a mouse model of AD.

## Introduction

The loss of cognitive function is the most devastating feature of Alzheimer's disease (AD) and is likely to involve a dysfunction of entorhinal-hippocampal circuitry [1], [2], [3], [4], [5]. The entorhinal cortex (EC) and hippocampus are among the brain regions where neurofibrillary tangles and amyloid-beta (Aβ) plaques first develop in AD patients [6], [7], [8], [9]. Functional and anatomic magnetic resonance imaging reveal a higher activation of hippocampal and EC circuits in patients with mild cognitive impairment compared to Controls, which is followed by a lower activity in AD [10]. The loss of synapses in EC-hippocampus network is also an important structural correlate of cognitive decline in AD patients at an early stage [11], [12]. In accordance with human *post mortem* data, synapse abnormalities and Aβ deposition in these two brain regions have been reported in animal models of Aβ overexpression [13], [14], whereas spontaneous nonconvulsive seizure activities in cortical and hippocampal networks of young APP transgenic mice have been documented [5], [15]. The more recent triple transgenic model of AD (3xTg-AD) also develops both neurofibrillary tangles and Aβ plaques in the EC and the hippocampus, without significant neuron loss [16], [17]. There are thus compelling arguments to hypothesize that the activity of entorhinal and hippocampal neurons is altered early in AD and is partly responsible for the first impairments in cognitive function.

Beneficial effects of docosahexaenoic acid (DHA) have been described in several transgenic animal models of AD [18], [19], including improved performance in the Morris water maze paradigm [20], [21], prevention of the hyperphosphorylation of tau [22], decreased Aβ levels [21], [22], [23], [24], [25] and protection from the loss of synaptic proteins [20], [26]. Most epidemiological prospective studies also support an association between higher DHA consumption and lower risk of developing age-related dementia (see discussion). Recent data from clinical assays reveal a potential nutraceutical role for DHA in preventing or ameliorating cognitive decline [27]. However, the effects of DHA on the physiology of cortical neurons within the EC-hippocampus loop, which could underlie these cognitive benefits, remain unexplored in animal models of AD.

The aim of this study was thus to investigate the beneficial effects of DHA in 3xTg-AD mice, an animal model of AD displaying both neurofibrillary tangles and Aβ plaques [17]. To establish functional correlates, we also studied the intrinsic and synaptic properties of EC deep layer neurons from NonTg and 3xTg-AD mice. We selected these neurons because they are key components of the entorhinal-hippocampal network [28], [29] while displaying a higher susceptibility to network excitation [28], [30]. Our results demonstrate that DHA intake alters intrinsic and synaptic properties of EC deep layer neurons, maintains cell membrane surface area, ameliorates object recognition and reduces the number of seizure-like akinetic episodes observed in 3xTg-AD mice.

## Results

### High DHA intake increased DHA and decreased arachidonic acid (AA) concentrations in the cortex of NonTg and 3xTg-AD mice

**Figure 1:**
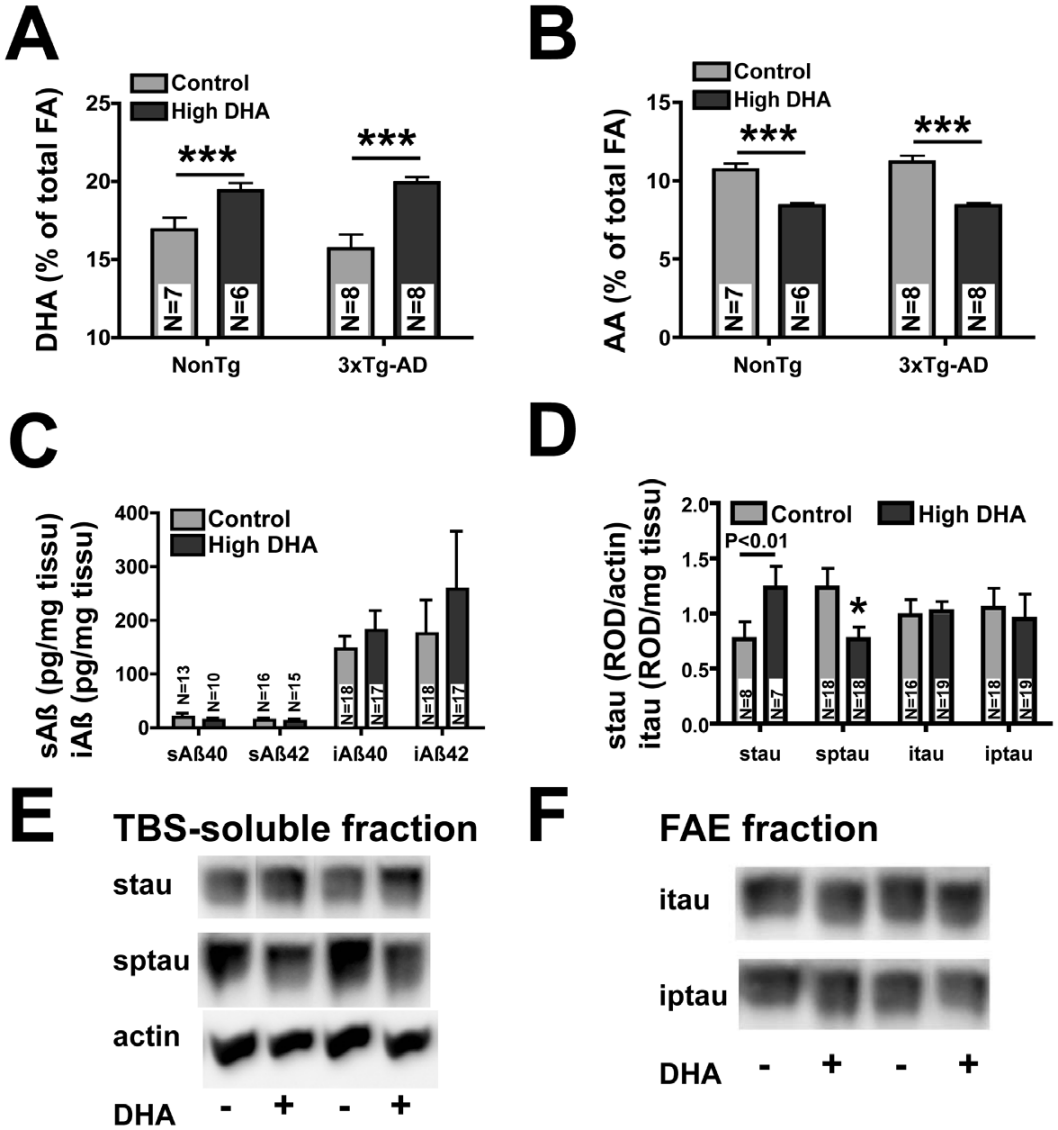
High DHA intake modulates the fatty acid profile of the frontal cortex of NonTg and 3xTg-AD mice and decreases soluble *p*tau. High DHA intake from 4 to 14 months of age increased DHA content (A) and decreased AA levels (B). (C) DHA consumption had no effect on the level of Aβ in soluble or insoluble fraction. (D) *p*tau was decreased in following DHA treatment, without any effect on total tau in soluble fraction and on tau (or *p*tau) in insoluble fraction. Illustrations of tau/*p*tau and actin bands in TBS-soluble fraction (E) and tau in FAE fraction (F). Values are expressed as mean ± SEM. Statistical comparisons were performed using two-way ANOVA and correlations were analyzed using a linear regression. Abbreviations: AA, arachidonic acid; DHA, docosahexaenoic acid; FA, fatty acid; FAE, formic-acid extract (insoluble fraction); iAβ, insoluble Aβ; itau, insoluble tau; i*p*tau, insoluble phospho-tau; ROD, relative optical density; sAβ, soluble Aβ; N, number of mice; stau, soluble tau; s*p*tau, soluble phospho-tau; TBS, tris-buffered saline (soluble fraction). *P<0.05, ***P<0.001

This study included 4 groups of 19 mice, of which 8 were used for electrophysiological and behavioral experiments. As reported previously [31], 3xTg-AD mice were heavier than NonTg mice (31% for mice fed with control diet and 16% for mice fed with high-DHA diet, P<0.001), whereas DHA intake had no effect on animal weight (P = 0.31, Table S1). Consistent with previous reports [20], [22], [32], high DHA consumption induced an increase in frontal cortex DHA of 15% in NonTg mice and of 27% in 3xTg-AD mice, compared to mice fed control diet (P<0.001, Figure 1A and Table S1). In parallel, mice fed a high-DHA diet had lower brain levels of AA (−22% for NonTg mice and −25% for 3xTg-AD; P<0.001, Figure 1B) and, therefore, a lower DHA/AA ratio (44% for NonTg and 68% for 3xTg-AD, P<0.001, Table S1). The DHA/AA ratio is important for essential neurobiological functions like neurotransmission and the equilibrium between PUFA metabolites involved in oxidative stress and inflammatory response [33], [34], [35]. Finally, we found an inverse relationship between AA and DHA concentrations (r^2^ = 0.61, P<0.001, data not shown).

### DHA treatment reduced the phosphorylation of TBS-soluble tau with no significant effects on Aβ

To determine whether DHA consumption altered the development of tau and Aβ pathologies, we quantified the concentration of tau (Western immunoblots) and Aβ (ELISA) in TBS-soluble and detergent-insoluble fractions from brain cortex homogenates (Table S2). We found no significant effects of DHA on Aβ pathologies (Figure 1C, Table S2), whereas DHA intake selectively decreased phosphorylated tau (*p*tau) in TBS fractions (P<0.05, Figure 1D-F, Table S2). The *p*tau/tau ratio was inversely correlated with the content of DHA in the frontal cortex (r^2^ = 0.26, P<0.05, data not shown). No significant effect on insoluble tau was observed.

### DHA improved mnesic performance deficit caused by Aβ/tau pathologies

**Figure 2:**
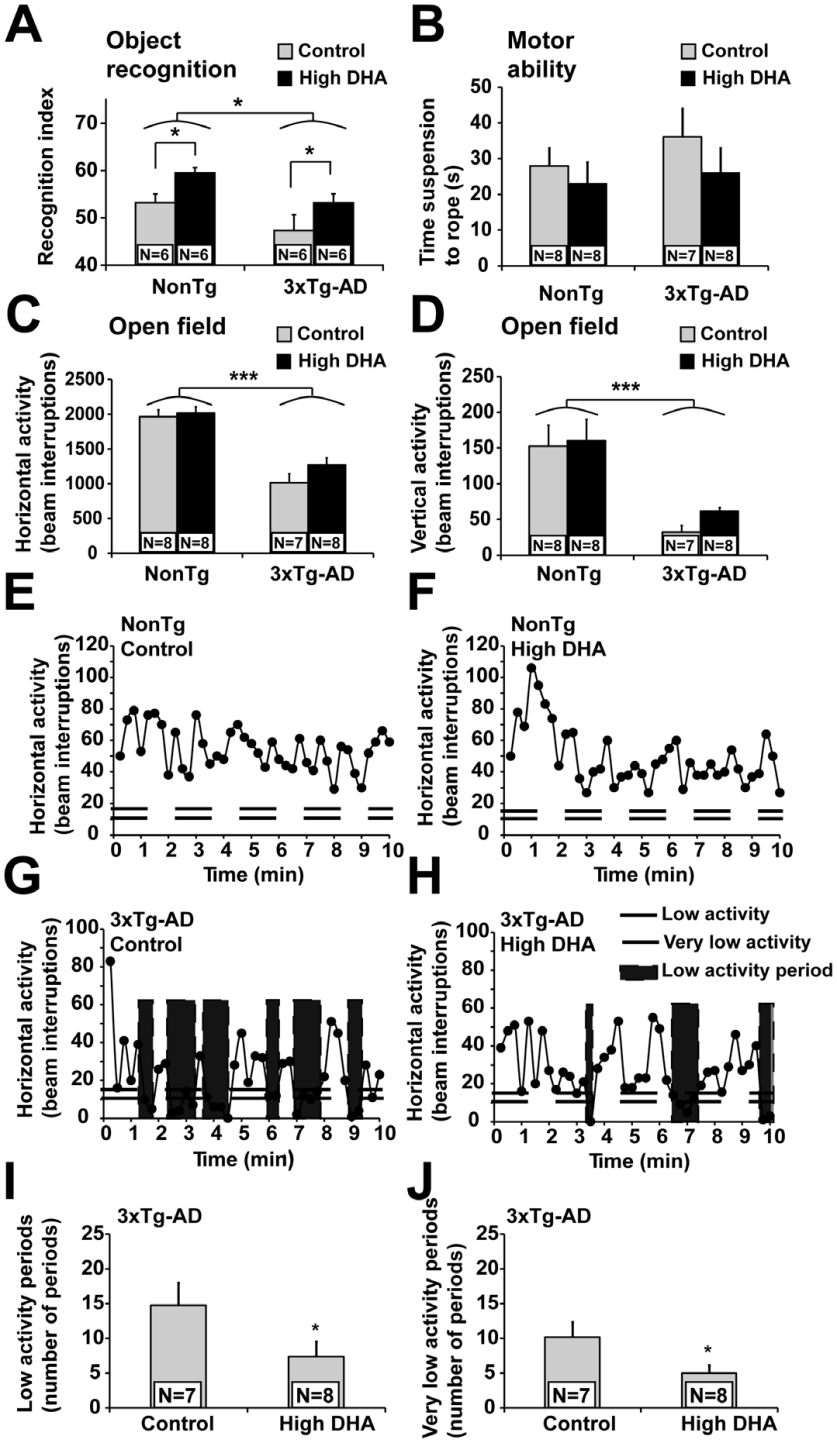
DHA prevents deficits in novel object recognition and episodes of low activity in mice 3xTg-AD. (A) Horizontal and (B) vertical locomotor activities were both decreased in 3xTg-AD mice compared to NonTg. (C) On the object recognition task, DHA intake improved the cognitive performance of NonTg and 3xTg-AD mice. Recognition index is represented by the percentage of time exploring the object 3 (100*object 3/total exploration during second test). (D) Forelimbs muscle tone necessary to cling onto a stretched cable was comparable between each group, indicating that motor problems did not account for the lower activity of 3xTg-AD mice. (G) 3xTg-AD mice displayed frequent periods of low activity (<15 beam interruptions per 15 sec) or very low activity (<10 beam interruptions per 15 sec), a feature not observed in NonTg mice (H). DHA treatment partly prevented the occurrence of (I) low activity periods and (J) very low activity periods in 3xTg-AD mice. Statistical comparisons were performed using two-way ANOVA or unpaired t-test (low and very low activity periods). Abbreviation: N, number of mice. P<0.05; ***P<0.001

The loss of cognitive/memory functions is the earliest devastating feature of AD. To evaluate this in 3xTg-AD mice, we used the novel-object recognition task. These experiments demonstrated that (1) 3xTg-AD performed significantly worse than NonTg mice (P<0.05) and (2) the consumption of DHA improved the recognition performance of both NonTg and 3xTg-AD mice (P<0.05) (Figure 2A, Table S4). These observations indicate that consumption of DHA improved the object recognition of 3xTg-AD mice, reaching performances comparable to NonTg mice fed with control diet.

The time spent in seconds to explore the object during the first exposition was 88±3 (n = 8) and 114±3 (n = 8) for NonTg mice fed with control or high-DHA diet, whereas it was 72±6 (n = 7) and 72±5 (n = 8) for 3xTg-AD mice fed with control or high-DHA diet. The adaptativity ratio (time spent exploring at 1 h divided by the time spent exploring during the conditioning phase) was 0.33±0.05 (n = 8) for NonTg mice fed with control diet, 0.35±0.03 (n = 7) for NonTg mice fed with high-DHA diet, 0.27±0.05 (n = 7) for 3xTg-AD mice fed with control diet and 0.22±0.05 (n = 8) for NonTg mice fed with high-DHA diet.

### DHA partly prevented the occurrence of akinetic episodes in 3xTg-AD mice

We next characterized the locomotor activity of animals in an open field during 10 minutes. We observed diminished horizontal (P<0.001) and vertical activities (P<0.001) in 3xTg-AD compared to NonTg mice (Figure 2C-D and Table S4), without a significant effect of DHA. The clinging test demonstrated an equal physical performance between each group, ruling out a defect in muscular ability as the main cause of decreased locomotor activity or exploratory behavior (Figure 2B). However, besides these gross alterations in locomotor activity, we observed peculiar discontinuities in the behavior of 3xTg-AD mice not present in NonTg animals. Indeed, 3xTg-AD mice exhibited frequent short episodes of low activity. These akinetic moments occurred spontaneously and they lasted several seconds (5–30 s). To quantify these akinetic episodes, we fragmented our recordings of locomotor activity into smaller, 15-sec periods and fixed two thresholds of low activity (Figure 2E–J). Periods of less than 15 or 10 beam interruptions were frequent in 3xTg-AD mice, strongly suggesting the occurrence of seizure-like freezing behavior. Interestingly, DHA intake reduced the number of akinetic episodes seen in 3xTg-AD mice (P<0.05, low and very low activity episodes, Figure 2I–J). In summary, our results indicate that the lower locomotor activity of 3xTg-AD mice may be explained by frequent akinetic episodes, a defect partly prevented by chronic DHA treatment.

### DHA alters passive properties of EC deep layer neurons and prevents the decrease of CC in 3xTg-AD mice

**Figure 3:**
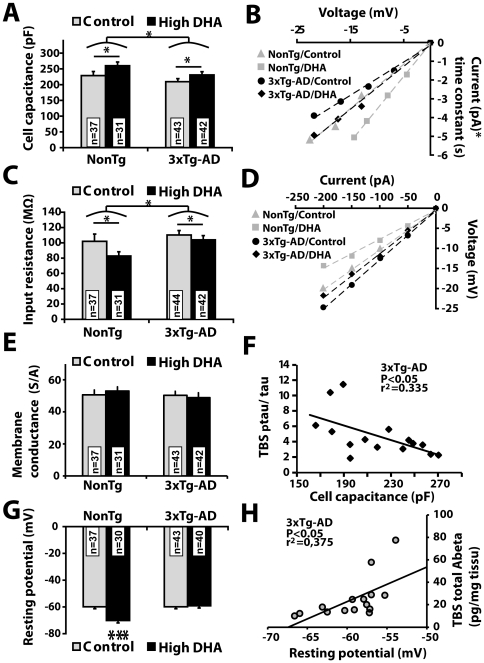
Effects of DHA intake on passive properties of EC deep layer neurons from NonTg and 3xTg-AD mice. (A) CC of EC neurons increased with DHA intake and decreased with 3xTg-AD expression. The slope of the injected current x time constant versus voltage variation plot was used to estimate CC. Typical examples of slopes are illustrated in panel B. (C) DHA decreased, whereas 3xTg-AD transgenes increased the input resistance following the injection of a hyperpolarized current. Input resistance was determined from the slope of the voltage variation versus injected current plot and examples of current-voltage slope from one cell per group are illustrated in panel D. (E) Membrane conductance was not modulated by diet or transgene expression. (F) Inverse relationship between cell capacitance and *p*tau in 3xTg-AD mice. (G) DHA intake hyperpolarized EC neurons by altering their resting potential from −60 mV to −70 mV, an effect only present in NonTg mice. (H) Positive relationship between resting potential and TBS-soluble total Aβ (Aβ40 + Aβ42) in 3xTg-AD mice. Values are expressed as mean ± SEM. Statistical comparisons were performed using two-way ANOVA (CC and input resistance) and one-way ANOVA followed by Tukey-Kramer posthoc test (resting potential; variable interaction). Correlation was performed using a linear regression. Recorded neurons were obtained from 8 mice per group. Abbreviations: CC, cell capacitance, n, number of recorded neurons. *P<0.05; ***P<0.001.

To identify underlying functional correlates of the behavioral effect of DHA, we next sought to determine whether DHA treatment alter the functional properties of EC neurons within the entorhinal-hippocampal circuitry (Figure S1). Indeed, EC neurons transmit information to the hippocampus [29], thereby playing an important role in memory [36], [37], [38]. We first studied intrinsic properties of EC neuron using patch-clamp recording in current clamp, focusing on passive electrophysiological parameters. We observed that CC, an indicator of total membrane area [39], [40], [41], was decreased in neurons from 3xTg-AD mice compared to NonTg (P<0.05; Figure 3A, 3B). DHA treatment prevented this decrease and had an overall increasing effect on CC in all animals (P<0.05). In contrast, IR was lower in 3xTg-AD neurons (P<0.05; Figure 3C and 3D) and higher in DHA-treated animals (P<0.05). The flux of ions crossing the membrane via ion channels contributes to total IR; increased movement of ions can lead to a lower resistance. Thus, the variation of IR observed here was likely a consequence of changes in CC since we found no significant difference in membrane conductance (Figure 3E). The strong correlation between IR and CC supports a tight link between the two passive properties of EC neurons (P<0.001 and r^2^ = 0.36 for NonTg mice, P<0.001 and r^2^ = 0.28 for 3xTg-AD mice). In addition, the relationship between CC and *p*tau cortical content suggests an association between tau pathology and membrane surface atrophy (Figure 3F). Overall, these observations strongly suggest that DHA increased the surface area of EC neurons, preventing the decrease observed in 3xTg-AD mice.

**Figure 4:**
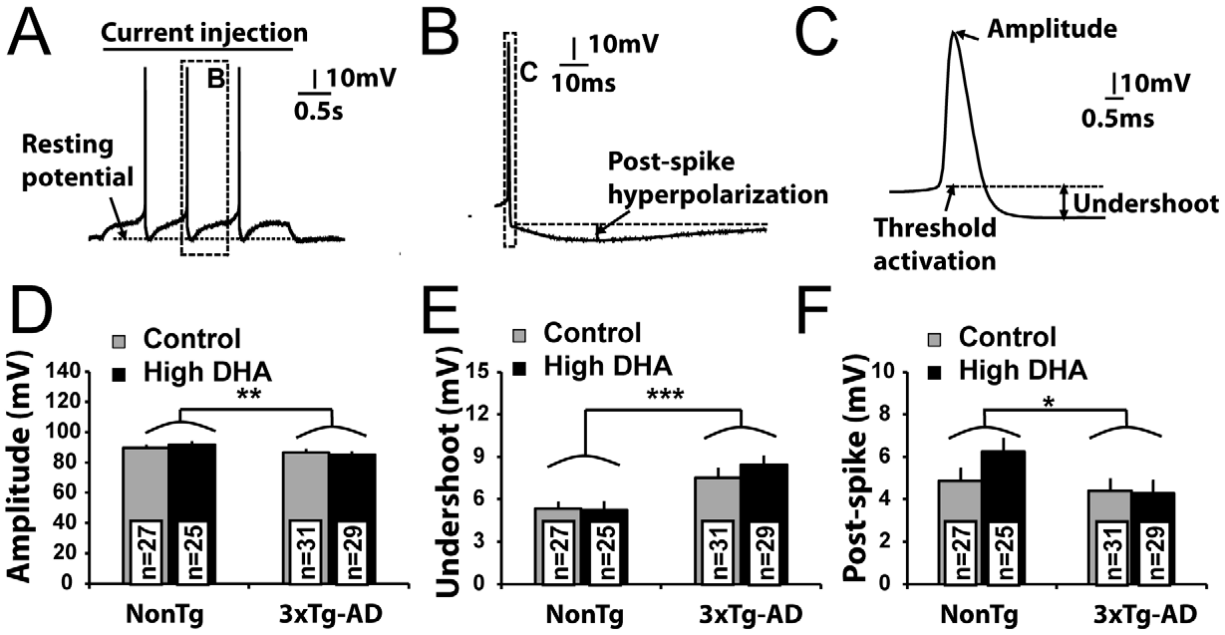
Alteration of action potentials characteristics in 3xTg-AD mice: no effect of DHA. (A) Example of entorhinal cortex (EC) neuron recording following an injection of a 3-s depolarizing current. In this typical trace, the injected current triggered three action potentials. (B) Representation of a post-spike hyperpolarization (zoomed from the dashed square in A). The post-spike hyperpolarization was calculated from the difference between the voltage undershoot after the action potential (dashed line) and the voltage peak of post-spike. (C) Representation of the voltage undershoot following the action potential (zoomed from the dashed square in panel B). Undershoot was the difference between the stabilized voltage after the action potential and the activation threshold. (D) Decreased amplitude was recorded in 3xTg-AD neurons, (E) increased voltage undershoot and (F) reduced post-spike hyperpolarization of EC neurons from 3xTg-AD mice. Values are expressed as mean ± SEM. Statistical comparisons were performed using two-way ANOVA. Recorded neurons were obtained from 8 mice per group. Abbreviation: n, number of recorded neurons. *P<0.05; **P<0.01; ***P<0.001.

In addition, we observed that DHA consumption led to the hyperpolarization of EC neurons of NonTg mice (P<0.001, Figure 3G), an effect not present in 3xTg-AD mice. Interestingly, the resting potential inversely correlated with DHA/AA concentration ratio (P<0.05, r^2^ = 0.42, data not shown) in NonTg mice and showed a positive relationship with TBS-soluble Aβ40 (P<0.05, r^2^ = 0.28, data not shown), TBS-soluble Aβ42 (P<0.05, r^2^ = 0.34, data not shown) and TBS-soluble total Aβ (P<0.05, r^2^ = 0.38, Figure 4H) in 3xTg-AD mice. Such positive relationship in 3xTg-AD mice between resting potential and Aβ concentrations (Figure 3H) suggests that accumulation of Aβ may have blocked the hyperpolarizing effect of DHA in 3xTg-AD mice. Consistent with a hyperpolarizing effect of DHA, we observed that the number of spontaneously active neurons in NonTg mice fed with DHA diet was approximately half of those from animal fed control diet (Table S6).

### Expression of transgenes, but not DHA intake, alters action potential characteristics

To further investigate implications of DHA or APP/PS1/tau transgenes in the physiology of entorhinal cortex deep layer neurons, we quantified key characteristics associated with a single action potential (Figure 4A, 4B and 4C, Table S3). Firstly, we observed a decrease in the amplitude of action potentials (P<0.01, Figure 4D), an increase of undershoot voltage variation (P<0.001, Figure 4E) and a reduction of post-spike hyperpolarization (P<0.05, Figure 4F) in 3xTg-AD neurons, compared to NonTg mice. DHA intake had no effect on these parameters. In summary, action potentials of 3xTg-AD EC neurons displayed important alterations.

### Increased firing activity of 3xTg-AD neurons: partial preventive effect of DHA

**Figure 5:**
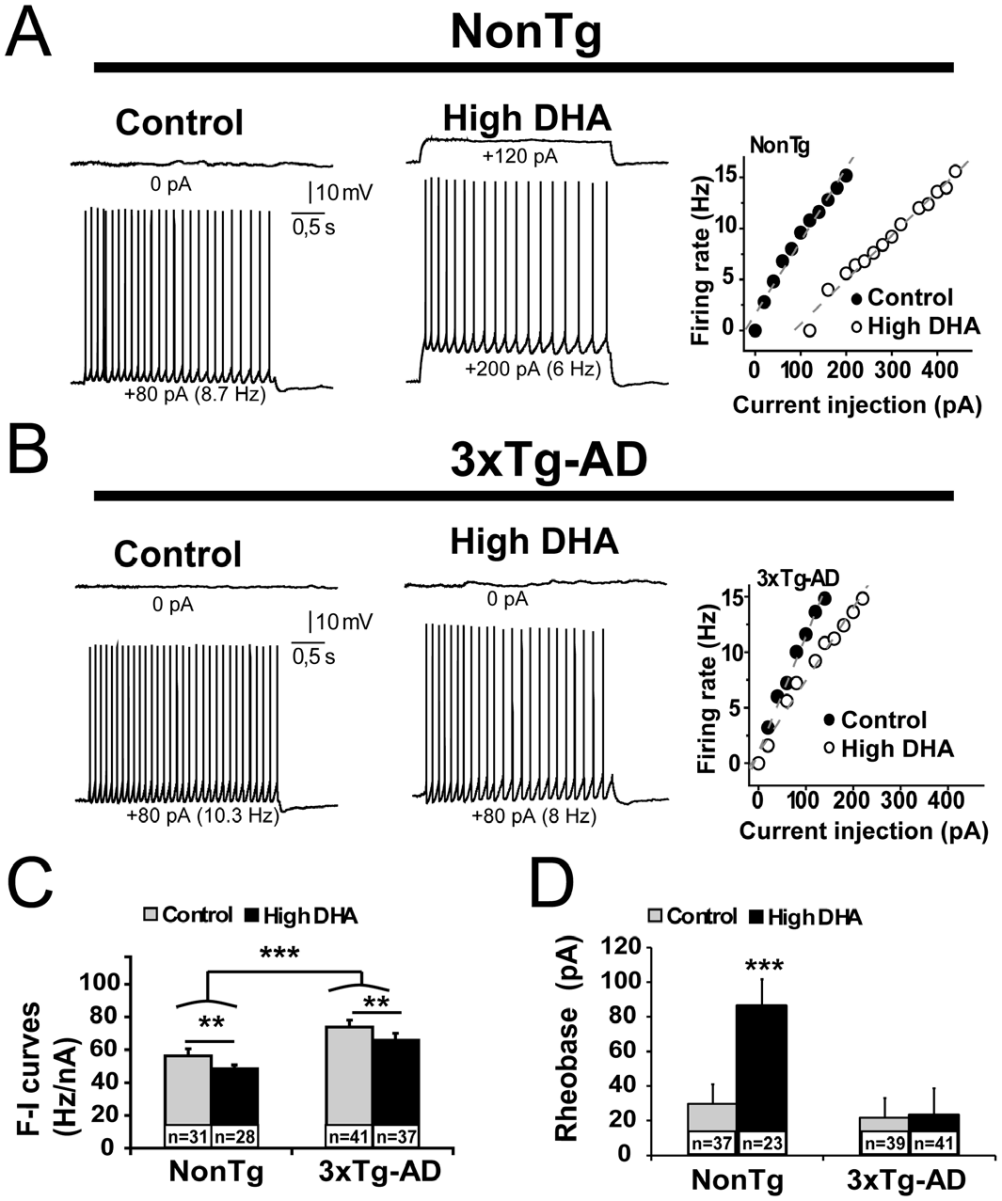
DHA treatment and genotype modulate active properties of EC deep layer neurons. (A, B) Examples of recording trace showing voltage response to a 3-s depolarizing current at the excitation threshold (top trace) and 80±5 pA above the rheobase (bottom trace) from the same neuron of each group. The robust increase in firing frequency detected in 3xTg-AD mice was partly prevented by DHA, which decreased firing frequency in all animals. The relationship between firing rate and injected current (F-I curves) from NonTg or 3xTg-AD neurons were illustrated in the graph at the right of the panel. (C) The steepness of F-I slopes was reduced by DHA intake whereas it was increased by transgene expression. (D) DHA intake increased rheobase only in NonTg mice. Abbreviations: F-I, firing rate versus injected current. Values are expressed as mean ± SEM. Statistical comparisons were performed using two-way ANOVA (F-I curves) and one-way ANOVA followed by Tukey-Kramer posthoc test (rheobase; variable interaction). Recorded neurons were obtained from 8 mice per group. Abbreviation: n, number of recorded neurons. **P<0.01; *** P<0.001.

To characterize the effect of DHA on firing properties of EC neurons, we next studied in current clamp two fundamental features of neuronal activity, (1) the F-I curves and (2) the intensity of depolarization required to deliver an action potential (rheobase). First, we found that the F-I curves were increased in 3xTg-AD mice (P<0.001), but decreased by DHA treatment (P<0.01, Figure 5A–C). This result indicates that firing activity of 3xTg-AD neurons was increased compared to NonTg neurons, whereas DHA had the opposite effects in all animals. We also found a negative relationship between CC and F-I curves (P<0.05 and r^2^ = 0.01, data not shown). Second, an increase of rheobase was detected in DHA-enriched neurons from NonTg mice (P<0.01, Figure 5D), whereas DHA had no effect on rheobase in 3xTg-AD animals. This is consistent with the hyperpolarizing effect of DHA on resting potential restricted to NonTg mice presented above, and further supported by a significant negative correlation between both parameters (P<0.001, r^2^ = 0.61). In summary, DHA consumption decreased the F-I curves of EC neurons through a CC-dependent mechanism, while Aβ/tau pathologies increased firing activity.

### Functional and molecular dysfunctions of glutamatergic synapses in 3xTg-AD mice

**Figure 6:**
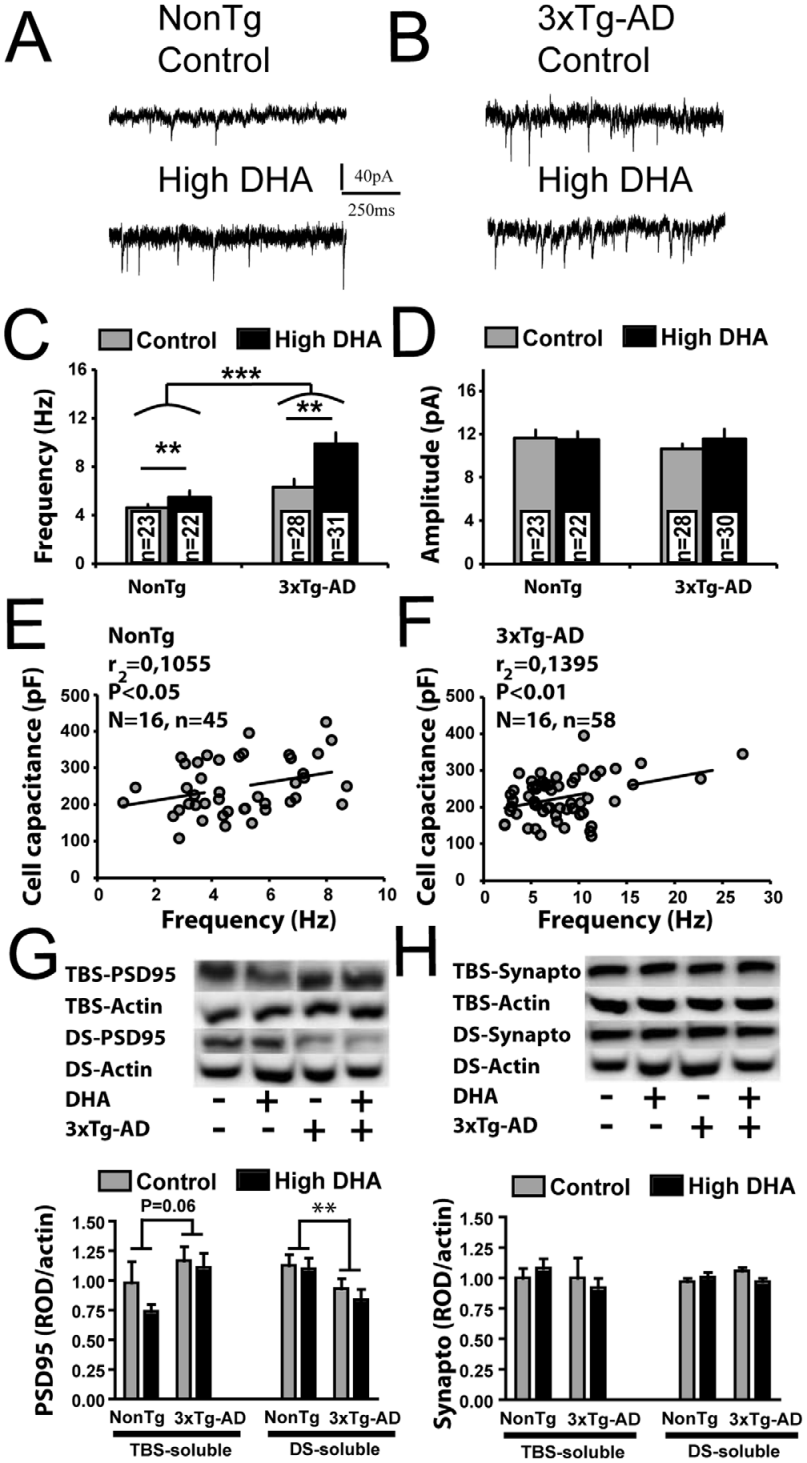
Functional and molecular impairments of glutamatergic synapses in 3xTg-AD mice. Cellular recordings of (A) NonTg or (B) 3xTg-AD EC neurons fed with the control or high-DHA diet. (C) Transgene expression as well as DHA intake increased the frequencies of sEPSC. (D) The mean amplitude of sEPSC did not differ between groups. CC was significantly correlated with the number of excitatory synaptic events EC neurons from (E) NonTg and (F) 3xTg-AD mice. (G) Postsynaptic protein PSD95 was decreased in detergent-soluble fractions from 3xTg-AD mice whereas DHA had no effect. (H) Diet and transgenes expression did not alter levels of synaptophysin, a presynaptic protein. Numbers of animals per group for molecular experiments were 18-19, except for TBS-soluble synaptophysin (n = 7–8/group). Statistical comparisons were performed using two-way ANOVA. Correlation analyses were performed using linear regressions. Abbreviations: DS, detergent-soluble; TBS, tris-buffered saline; ROD, relative optical density; sEPSC, spontaneous excitatory post-synaptic current. Recorded neurons were obtained from 8 mice per group. Abbreviations: n, number of recorded neurons; N, number of mice. **P<0.01; *** P<0.001.

Cerebral hyperactivity and defective network activity were reported in APP transgenic models of AD [5], [15] and AD patients [42], [43], [44]. Moreover, molecular impairments within glutamatergic synapses were found in AD patients and mouse models of AD [2], [20], [26], [45], [46]. To further investigate synaptic dysfunctions in 3xTg-AD mouse, we measured the sEPSC of EC neurons. First, we found that 3xTg-AD neurons displayed more sEPSC than NonTg (Figure 6A–C, P<0.001), suggesting the presence of a persistent hyperactivity of glutamatergic synapses in EC from 3xTg-AD mice and consistent with a network dysfunction. Interestingly, DHA treatment did not prevent synaptic hyperactivity of EC neurons, but rather increased the frequency of sEPSC in all animals (Figure 6C, P<0.01). Amplitude of sEPSC was not modulated by dietary DHA and expression of transgenes, suggesting that the depolarizing strength of each synapse remained unchanged for all groups (Figure 6D). The higher frequency of sEPSC is likely be related to the ability of DHA to increase neurite outgrowth [47] and dendritic spine density [48], [49], which would promoting synapse formation, thereby increasing detection of sEPSC [50]. The increase of DHA-induced CC and the significant correlation between CC and sEPSC in both genotypes support the latter idea (Figure 6E–F, P<0.05 and r^2^ = 0.10 for NonTg P<0.01 and r^2^ = 0.13 for 3xTg-AD). To assess possible molecular correlates, we quantified the level of synaptophysin and PSD95 proteins, respectively pre- and postsynaptic markers of glutamatergic synapses [51]. PSD-95 is involved in the regulation of the ratio of excitatory versus inhibitory presynaptic contacts through neuroligin-dependent pathways, suggesting that PSD-95 is involved in network activity [52], [53]. The apparent translocalisation of PSD-95 from membrane to cytosolic fractions (Figure 6G) observed here might thus be a compensatory mechanism resulting from the hyperactivity of glutamatergic synapses. Such loss of PSD-95 is consistent with previous analyses in the membrane fraction from the cortex of AD patients or Tg2576 mice [20]. On the other hand, absence of synaptophysin alteration in 3xTg-AD mice (Figure 6H) is consistent with report on other animal model of AD [20], [54] and suggest that the rise of sEPSC was not related to a massive change in synapses number. DHA intake had no effect on both pre- and postsynaptic markers.

### Acute application does not mimic the effect of chronic DHA treatment

**Figure 7:**
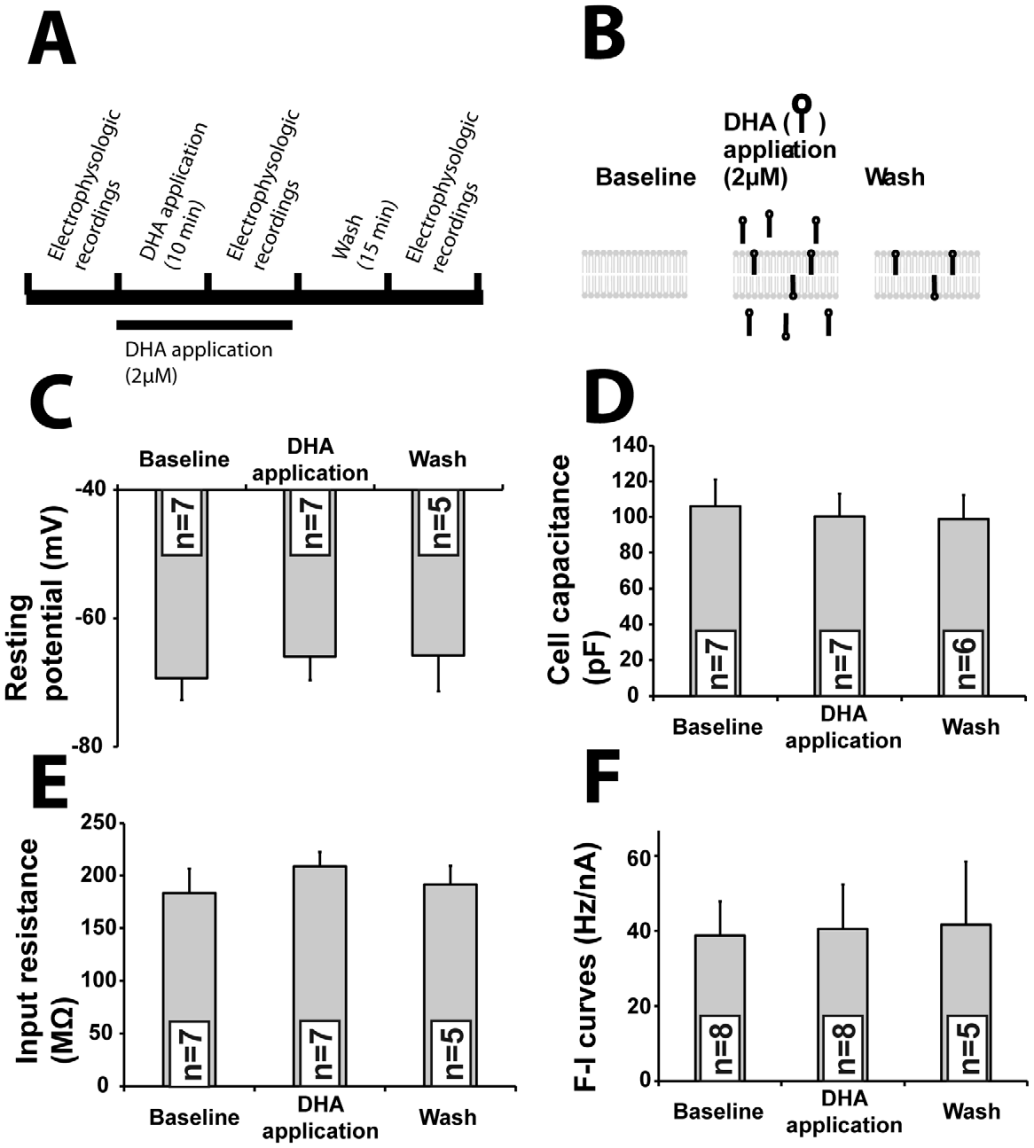
Acute administration of DHA does not replicate the chronic effects of DHA treatment. (A, B) Timeline and schematic view of DHA application experiments on EC neurons on brain slice. (C, D, E, F) In contrast to chronic oral treatment, acute DHA application on slice had no effect on (C) resting potential, (D) CC, (E) input resistance and, (F) F-I curves. Statistical comparisons were performed using paired t-test. Abbreviations: CC, cell capacitance; F-I, firing rate versus injected current. Recorded neurons were obtained from 5 mice. Abbreviation: n, number of recorded neurons.

We then verified whether an acute exposure to DHA could reproduce the effects of the chronic DHA treatment. *In vitro* or *in vivo* data show that DHA incorporates into cell membrane phospholipids within seconds [55], [56], [57]. We thus incubated brain slices with DHA during 10 minutes followed by the same patch-clamp experiments described above (Figure 7A–B and Table S5). Previous studies have shown that DHA incorporates within seconds into membrane phospholipids, where its distribution reaches equilibrium within 10 minutes [55]. We found that acute application of DHA did not alter the resting potential (Figure 7C), CC (Figure 7D), input resistance (Figure 7E) or F-I curves (Figure 7F). These experiments strongly suggest that the effects of DHA treatment described above were not caused by binding to an ionic receptor or by modifying membrane properties. Rather, long-term mechanisms such as morphology alteration, gene transcription or long-term consequence of lipid profile modification (such as a decrease in AA and its derivatives) are likely to be necessary to explain the chronic effect of DHA on passive and active properties of EC deep layer neurons.

## Discussion

Neuronal network dysfunction within the EC - hippocampus loop has been hypothesized to underlie cognitive dysfunction in AD. The results presented here indicate that EC neurons from mice genetically programmed to develop extensive Aβ and tau pathologies display alterations of intrinsic properties consistent with reduced membrane surface (atrophy) and show increased neuronal activity. The effects of DHA intake on classical markers of AD neuropathology in 3xTg-AD mice were limited to a decrease of phosphorylated tau, in accordance with a previous report using the same transgenic animals fed during a similar amount of time, but with almost twice the daily dose of DHA [22]. On the other hand, DHA exerted powerful protective effects against alterations of intrinsic properties and deterioration of cognitive performance in 3xTg-AD mice.

### Defects in intrinsic properties of EC neurons in 3xTg-AD mice: protective effect of DHA against membrane surface atrophy

Neuronal atrophy and reduction in synapses and/or dendrites are well-recognized features of AD, which have been often found in animal models as well [12], [14], [58], [59]. Since cellular atrophy is associated with decreased cell surface, we quantified membrane surface area of EC neurons, using electrophysiological measurement of CC [39], [40], [41]. Therefore, the loss of CC in 3xTg-AD mice observed here is likely attributed to a diminution of cellular surface. Conversely, chronic intake of DHA increased CC in all animals, thereby fully correcting the decrease detected in 3xTg-AD mice. Such a hypertrophic effect of DHA is in agreement with studies reporting that DHA increases the density of synaptic spines *in vivo* [48], [49] and stimulated the growth of neurites *in vitro* [60], an effect attributed to binding to syntaxin-3 [61]. Expected consequence of such DHA-induced morphologic modulations would logically include enhanced glutamatergic synaptic activity and increase sEPSC frequency as observed here in EC neurons and reported in embryonic neuronal cultures [50]. Such increases of CC and sEPSC frequency could lead to a better network integration of DHA-treated neurons, enabling them to collect and process a larger flow of information.

### Neuronal hyperactivity in the 3xTg-AD mouse model: complex action of DHA

In recent years, mounting evidence support the hypothesis that defective neuronal activity plays an important role in the development of symptoms in early AD [4], [5], [44]. Functional magnetic resonance was used to detect an hyperactivity of EC and hippocampus during the early stages of the disease, before full blown neurodegeneration occurs [10]. Hyperactive episodes akin to non-convulsive epileptic seizures were clearly demonstrated in a mouse model of AD [5], [15]. Neuronal activity and the formation of Aβ peptides are part of a vicious circle evidenced in organotypic culture [62], whereas populations of hyperactive neurons surrounding senile plaques were evidenced with calcium imaging in animal models [63].

However, little is known about cellular mechanisms leading to such cerebral hyperactivity in AD. Our study highlights potential mechanisms deriving from intrinsic properties of neurons. Most importantly, an increase in firing activity was recorded in 3xTg-AD neurons, for which at least two explanations can be proposed. On one hand, the loss of surface area, as suggested by lower CC, is likely to have altered current dispersion throughout the membrane of 3xTg-AD neurons. Hence, a fixed depolarizing input produces a higher current density and voltage variation in smaller neurons, as schematized in Figure S2 and previously shown in modelized motoneurons [64]. On the other hand, the action potential modifications observed in 3xTg-AD neurons could contribute to increase their firing rate. Indeed, the lower amplitude of action potential recorded in 3xTg-AD EC neurons may be the results of an increase in the voltage undershoot. Such key modifications in action potential are known to reduce calcium-dependent potassium current and, consequently, enhance firing activity [65], [66], [67], [68], [69]. The reduction of post-spike hyperpolarization, a calcium-dependent current [68], [70] observed in 3xTg-AD animals, support this idea.

The effect of DHA on neuronal activity was more complex. In NonTg animals, DHA induced hyperpolarization of EC neurons, consistent with reported antiarrhythmic and anticonvulsant effects of DHA [71], [72], [73], [74], [75]. Accordingly, DHA was also found to increase the rheobase in NonTg mice. Overall, these observations support the idea that DHA downregulates neuronal activity. However, the effect of DHA on resting potential or rheobase were not observed in 3xTg-AD mice. Similar alterations of resting potential were reported in neocortical cells/dentate granule cells of APP/PS1 mice model of AD [15] and in vitro Aβ application was known to depolarize neuron [76], [77], suggesting a key role of Aβ pathology in alteration of resting potential. In addition, DHA did reduce firing activity following sustained activation (F-I curves), but not sufficiently to fully prevent the increase seen in 3xTg-AD mice. Here again, altered dispersion of injected current on larger DHA-enriched neurons is a compelling explanation for the decreasing effect of DHA on F-I curves [64]. The correlation between CC and F-I curves stands in agreement with this proposition. In summary, DHA may reduce neuronal activity through hyperpolarization of the cell membrane and by reducing the firing activity in NonTg mice. However, these effects of DHA were partly blunted by Aβ/tau pathologies and not sufficient to fully prevent hyperactivity of EC neurons in 3xTg-AD mice.

At the synaptic level, we observed a rise of sEPSC frequency in 3xTg-AD mice, without any concurrent change in synaptic input indexed with synaptophysin. This indicates higher spontaneous activity of afferent excitatory synapses, consistent with EC hyperactivity. The lower rheobase and the steeper F-I curves in 3xTg-AD neurons suggest an involvement of neuronal activity in the observed synaptic hyperactivity. Reciprocally, synaptic hyperactivity is known to increase glutamate-dependent conductance, resulting in cell depolarization and an increase of F-I curves [78], [79], [80], [81]. Such a synergy between synaptic and intrinsic properties may favor unrestrained brain activity and thus provides an interesting explanation for the hyperactivation of the entorhinal-hippocampal circuitry observed in the early stages of AD.

### DHA modulates intrinsic properties of EC neurons: cognition enhancing and antiseizure activities?

It is well known that EC neurons transmit cerebral information to the hippocampus [29], a region strongly involved in memory and cognition. The defects in novel object recognition of 13-month-old 3xTg-AD mice observed here is consistent with at least two previous reports [82], [83]. Chronic dietary treatment with DHA improved object recognition and 3xTg-AD mice treated performed as well as NonTg animals on control diet. Such protective action of DHA against deterioration of cognition is in agreement with previous studies in APP mouse models of AD, which develop only Aβ pathology [20], [21], and with clinical trials on cognitive impairment associated with aging.

Indeed, recent clinical assays report improvement of cognitive performance following n-3 PUFA intake, in cognitively normal or individuals with mild AD or MCI [27], [84], [85], [86], but not in moderate or advanced AD [84], [85], [86], [87], [88], [89]. In addition, our data is consistent with most case-control and longitudinal observational studies based on reported food consumption or blood fatty acid measurements, which support an association between consumption of fish and a lower risk of developing dementia [90], [91], [92], although no such association was detected in two large cohorts [93], [94].

One of the most interesting observations reported here are the frequent episodes of low activity recorded specifically in 3xTg-AD mice. These short sequences of low activity likely have a central origin not caused by motor deficits, as 3xTg-AD and NonTg mice performed similarly in grip strength test. Seizure-like activity has been previously evidenced in animal models of AD, mainly based on EEG readouts and calbindin-D28k immunoreactivity [5], [15]. These anomalies were specifically associated with abnormal network excitability and remodeling of inhibitory circuits in the hippocampus-cortical loop [5]. Reminiscent of the present observations, Palop and al. reported that during EEG-confirmed seizures, mice were immobile and subsequently resumed their exploratory behavior [5]. The higher spontaneous activity of excitatory synapses found in the entorhinal cortex of 3xTg-AD mice is consistent with the contention that cerebral hyperactivity coincides with the observed freezing behavior. Most interestingly, chronic DHA treatment significantly reduced the occurrence of these episodes of low activity in 3xTg-AD mice, consistent with previous evidence of anti-seizure action of DHA [71], [72], [73], [74], [75]. Since remodeling of neuronal circuits in the hippocampus may be instrumental in the neuronal network abnormalities seen in APP mice [5], the regulating effect of DHA on CC and firing activity in EC neurons may underlie its potential preventive effect of DHA on cognitive decline and akinetic episodes observed here in the 3xTg-AD mouse.

### Conclusion

In summary, our data show that 3xTg-AD mouse exhibited poor performance in object recognition task and frequent episodes of low activity akin to a seizure-like freezing behavior. These cognitive defects were associated with series of alterations in passive, active and synaptic properties of EC neurons, including (i) reduced CC, (ii) increased firing activity and (iii) enhanced sEPSC frequencies. Chronic treatment with DHA exerted a full, or at least partial, preventive action against these electrophysiological and behavioral defects, except on the basal hyperactivity of EC neurons. Overall, our results show that chronic DHA treatment has a direct effect on neuronal function, which is highly relevant to its cognition-enhancing properties and potential therapeutic effects against epilepsy and AD.

## Methods

### Ethics statement

The use of animals was approved by the Laval university animal ethics committee (approval ID  = 07-113 and 07-061) and all procedures were performed according to the guidelines of the Canadian Council on Animal Care.

### Animals and diets

3xTg-AD mice have been previously described [17]. Briefly, the 3xTg-AD mice express the mutated gene PS1_M146V_ (knockin) and mutated human genes APP_Swe_ and tau_P301L_ in the same locus, both under the control of the mouse Thy1.2 regulatory element [17]. NonTg mice were derived from the original mouse line with the same genetic background. Only NonTg mice and homozygous 3xTg-AD were used in this study. Mice were exposed to two isocaloric diets: (i) a control diet containing no long chain n-3 PUFA (DHA or eicosapentaenoic acid (EPA)) but 7 and 67 µmole/g of α-linolenic (LNA, 18∶3 n-3) and linoleic (LA, 18∶2 n-6) acid, respectively; or (ii) a high-DHA diet with 9.5, 0.5 and 34 µmole/g of DHA, LNA and LA, respectively. The n-6∶n-3 PUFA ratios were of 10.4 and 2.8 in the control and high-DHA diets. The concentration of DHA in the high-DHA diet corresponded to a daily dose of approximately 0.6 g kg^-1^ day^-1^ (assuming a 25 g mouse eats 3 g of food per day). The source of DHA was a microencapsulated formulation (MEG-3) of fish oils from Ocean Nutrition Inc (Halifax, Canada) in order to protect DHA molecules from oxidation [95], [96], [97]. MEG-3 contains a DHA:EPA ratio of at least 4∶1 both in a triglyceride form. The main antioxidant in MEG-3 is vitamin C (5% w/w). The quantity of vitamin C supplemented to animals treated with MEG-3 (2.25 mg/kg) is lower that vitamin C normally added in all diets (5 mg/kg) and to vitamin C endogenously produced by the mouse. Indeed, the mouse can synthesize endogenous vitamin C through conversion of glucose into vitamin C in the liver by L-gulonolactone-gamma-oxidase [98], [99]. Thus, it is not even a vitamin for the mouse and a minimal addition of vitamin C in the diet is not expected to significantly alter their natural baseline levels. Importantly, the two diets were exactly the same in terms of total fat (5% w/w), total carbohydrates (66% w/w), total protein (20% w/w), total calorie (4 kcal/g), fibers, vitamins, minerals, and antioxidants (except for vitamin C in MEG-3). Moreover, both of these diets did not contain phytoestrogens, which are present in unpredictable amounts in most laboratory chows and are known to influence the hormonal status by acting as partial estrogen agonists [100], [101]. The formula of these purified diets (produced in collaboration with Dr Matthew Ricci from Research Diets Inc., New Brunswick, NJ) has been precisely determined to avoid any batch to batch variations. The dietary treatment started at 4 months of age and lasted until the mice were sacrificed between 12 and 14 months of age (13.2±1.6 months) for biochemical analysis (up to 19 mice per group) and electrophysiological experiments (8 mice per group). This age range was chosen to avoid any interaction with developmental or maturation processes in younger ages and to gather endpoints at an age when Aβ/tau pathology and cognitive deficits are readily detectable. To perform biochemical analysis, animals were perfused with PBS buffer (pH 7.2) containing protease inhibitors (1 tablet/200 ml; Sigma, St.Louis, MO). Brain regions were dissected from left hemisphere. Frontal cortex (∼20 mg) was used to estimate brain content of fatty acids whereas the rest of the cortex (parietal, temporal and occipital cortex) was pooled for biochemical measurements.

### Preparation of tissue samples

After adding 8 volumes of Tris-buffered Saline (TBS) containing Complete™ protease inhibitors cocktail (Roche, Indianapolis, IN), 10 µg/ml of pepstatin A, 0.1 mM EDTA and phosphatase inhibitors (1 mM each of sodium vanadate and sodium pyrophosphate, 50 mM sodium fluoride), frozen samples were sonicated briefly (3×10 s) and centrifuged at 100,000× g for 20 min at 4°C to generate a TBS-soluble intracellular and extracellular fraction (soluble fraction). The TBS-insoluble pellet was sonicated in 5 volumes of lysis buffer (150 mM NaCl, 10 mM NaH_2_PO_4_, 1% Triton X-100, 0.5% SDS, and 0.5% deoxycholate) containing the same protease and phosphatase inhibitor cocktail. The resulting homogenate was centrifuged at 100,000× g for 20 min at 4°C to produce a lysis buffer-soluble fraction (detergent-soluble fraction). The pellets (detergent-insoluble fractions) were homogenized in 175 µl of 90% formic acid followed by a short sonication (3×10 s). The resultant suspension was centrifuged (15,000× g; 4°C; 20 min) and 20 µl of the supernatant was neutralized with 1∶13 dilution of Tris-base 2 M (pH 10) to be used for ELISA (see below). The rest of the supernatant was dried out by SpeedVac (Thermo Savant, Waltham, MA), solubilized in Laemmli's buffer and processed for Western immunoblotting [31].

### ELISA

Insoluble Aβ40 and Aβ42 were measured by the Aβ [1]–[40] and [1]–[42] ELISA kits (Biosource, Camarillo, CA). Soluble Aβ40 (kit II) and Aβ42 (kit high-sensitive) were measured using Human Aβ ELISA kits from WAKO (Osaka, Japan). The two ELISAs were performed according to manufacturer's recommendations and the plates were read at 450 nm using a Synergy™ HT multi-detection microplate reader (Biotek, Winooski, VT) [31].

### Western immunoblotting

For Western immunoblotting [31], protein concentration was determined using bicinchoninic acid assays (Pierce, Rockford, IL). Equal amounts of protein per sample (15 µg of total protein per lane) were added to Laemmli's loading buffer, heated to 95°C for 5 min before loading, and subjected to sodium dodecyl sulfate-polyacrylamide gel electrophoresis. Proteins were electroblotted onto PVDF membranes (Millipore, Billerica, MA) before blocking in 5% nonfat dry milk and 1% bovine serum albumin (BSA) in PBS-Tween 20 for 1 h. Membranes were immunoblotted with appropriate primary and secondary antibodies followed by chemiluminescence reagents (Lumiglo Reserve, KPL, Gaithersburg, MD). Band intensities were quantified using a KODAK Image Station 4000 MM Digital Imaging System (Molecular Imaging Software version 4.0.5f7, Carestream Health, Rochester, NY). The following antibodies were used in this study: anti-actin (ABM, Richmond, Canada), anti-CAMKII (Stressgen, Victoria, Canada), anti-cofilin (Cell signaling technology, Boston), anti-Drebrin (MBL International, Woburn, MA), anti-group IV iPLA2 (Santa Cruz Biotechnology, Santa Cruz, CA), anti-PAK1 (Invitrogen, Ontario, Canada), anti-PAK3 and anti-total PAK (Cell signaling technology, Danvers, MA), anti-phospho PAK: P141 (BioSource, Camarillo, CA), anti-PSD-95 (Millipore, Billerica, MA), anti-SNAP-25 (Covance, Princeton, NJ), anti-synaptophysin (Millipore, Billerica, MA), anti-syntaxin 3 (Sigma, St. Louis, MO), anti-tau-13 (Covance, Princeton, NJ), anti-tau CP13 (phosphorylated at serine 202/threonine 205, gift from Dr Peter Davies, Albert Einstein College of Medicine, New York, NY).

### Lipid extraction and gas chromatography

Since the incorporation of dietary DHA is similar in different regions of the cortex [102], [103], [104], [105], lipid profiles were determined only in the frontal cortex. Experiments were performed as previously described [31], [32]. Approximately 20 mg of frozen frontal cortex from each mouse was used for lipid extraction. Weighed brain tissues were homogenized with BHT-Methanol (Sigma, St. Louis, MO) and two volumes of chloroform (J.T. Baker, Phillipsburg, NJ) and 0.5 volume of NaH_2_PO_4_ (0.2 M)-buffer solution were added to the resulting homogenate. After centrifugation at 3500 RPM, the lower layer (chloroform fraction) was collected [106]. Lipid extracts were transmethylated with BF_3_-MeOH (Alltech, State college, PA) at 100°C for 60 min. After cooling down, 2 ml of water and 2 ml of hexane (J.T. Baker) were added. After homogenization and centrifugation at 3500 RPM, the upper layer (hexane fraction) was collected, dried down to about 100 µl and transferred to a gas chromatography autosampler vial and capped under nitrogen. Fatty acid methyl esters in brain tissue were quantified on a model 6890 series gas chromatograph (Agilent Technologies, Palo Alto, CA) using a FAST-GC method. One microliter of each sample was injected at a 25∶1 split ratio. Peak identification of fatty acid methyl esters was performed by comparison to the peak retention times of a 28-component methyl standard (Nu-Chek Prep, Elysian, MN) [107].

### Slice preparation for electrophysiology recordings

Horizontal brain slices of NonTg (8 controls and 8 high DHA) and 3xTg-AD (8 controls and 8 high DHA) mice were prepared from the right hemisphere as previously described [108], [109]. Briefly, mice were deeply anaesthetized with ketamine (100 mg/kg, ip) and xylazine (10 mg/kg, ip), and decapitated. The brain was removed quickly (*<*60 s) and placed in an ice-cold solution containing (mM): 210 sucrose, 3.0 KCl, 1.0 CaCl_2_, 3.0 MgSO_4_, 1.0 NaH_2_PO_4_, 26 NaHCO_3_ and 10 glucose, saturated with 95% O_2_ and 5% CO_2_. Horizontal slices of 300 µm were cut from inferior to superior brain with a vibrating tissue slicer (VT 1000 s, Leica, Wetzlar, Germany), and kept in artificial cerebral spinal fluid (ACSF) containing (mM): 124 NaCl, 3.0 KCl, 1.5 CaCl_2_, 1.3 MgCl_2_, 1.0 NaH_2_PO_4_, 26 NaHCO_3_ and 20 glucose, saturated with 95% O_2_ and 5% CO_2_ at room temperature (21–23°C). Slices were allowed to recover for at least 1 h before recording.

### Patch-clamp recording

For recording, a slice was transferred to a submerge-type chamber and continuously exposed to ACSF heated to 30–32°C, saturated with 95% O_2_ and 5% CO_2_ and flowing at a rate of 2.0±0.2 ml/min. When mentioned, DHA (Cayman Chemical Compagny, Michigan City, IN) at a concentration of 2 µM was added to the extracellular solution. This concentration of DHA is under its critical concentration for micelle formation (CMC) [110], [111], [112]. DHA in solution achieves equilibrium into the membranes phospholipid within 10 minutes [55], [113] by an adsorption mechanism [114].The slices were viewed first with a 4x objective and deep layer of EC was located beside hippocampus (Figure 1). In general, two slices could be recorded per hemisphere. Large deep layer neurons in the EC were then viewed under near-infrared illumination with a 40x water-immersion objective (Fluor, 40x, 0.80W, Nikon, Mississauga, ON, Canada) and a CCD camera (IR-1000, MTI, Michigan City, IN).

Experiments were conducted at 30–32°C. Patch pipettes were pulled from thick wall borosilicate glass (1.5/0.84 mm, WPI, Sarasota, FL) on a horizontal puller (P-97, Sutter Instruments, Novato, CA). The pipette solution contained (mM): 100 KMeSO_4_, 15 KCl, 4 ATP-Mg, 10 creatine phosphate, 10 HEPES, 0.5 EGTA (pH 7.2 with KOH). Electrodes had resistances between 2 and 5 MΩ. Liquid junction potential, estimated to be +5 mV, was not corrected. The seal resistance was greater than 2 GΩ. Whole-cell recordings were made at the soma with a Multiclamp 700A amplifier (Molecular devices, Sunnyvale, CA). The serial resistance, usually between 7 and 20 MΩ, was compensated using the bridge balance in current-clamp and was not compensated in voltage-clamp. Experiments were conducted using the Axograph 4.9 program (Molecular devices). Data were digitized at 8 or 16 kHz and were not filtered or filtered at 1 kHz, depending on the recording protocol.

### Selection of neurons and data analysis for electrophysiology experiments

Among 186 recorded cells (49 NonTg/control, 34 NonTg/high-DHA, 52 3xTg-AD/control, 51 3xTg-AD/high-DHA), 167 neurons had sustained spiking (41 NonTg/control, 32 NonTg/high-DHA, 47 3xTg-AD/control, 47 3xTg-AD/high-DHA) whereas the rest of neurons had non-sustained spiking. To study neurons with the same electrophysiologic properties, we analyzed only neurons having a sustained spiking. AxoGraph 4.9 and Origin 7 (OriginLab, Nothampton, MA) software programs were used for analysis. Passive and active properties were tested in I-clamp, whereas spontaneous excitatory postsynaptic currents (sEPSC) were quantified in V-clamp with a voltage clamp to −60 mV. The firing rate was estimated by counting the number of spikes during the step and the result was plotted versus the amplitude of the injected current (F-I graph). The slope (F-I curve) was calculated for the firing frequencies included between 0 and 15 Hz by using linear regression analyses. The rheobase was estimated graphically on the F-I graph. Action potentials were detected using the event detection package of the AxoGraph and the results were analyzed with Origin 7. The action potential was characterized in the last 1.5 second and the threshold was measured using a cursor, by inspecting a 5 ms segment around the rising phase of the action potential. The input resistance (R) was estimated from the graph slope of voltage variation (V) versus hyperpolarizing current injection (I). The calculation was derived from the formula “ V  =  R * I ”. The injected current duration was 400 ms and hyperpolarized current amplitudes were 50, 100, 150 and 200 pA. The cell capacitance (CC) was estimated by the formula “ I * Τ  =  CC * V ” (i.e. 1st order RC circuit), where Τ is the time constant of voltage variation (measured by fitting a single exponential function). CC corresponds to the linear slope of the graph displaying the relationship between “ I * T ” and “ V ”. We measured CC in I-clamp because this recording mode generated more accurate values than V-clamp [41]. *The membrane conductance (Gm) was calculated from the resistance and CC of the neuron using the formula* “ Gm  =  R^−1^ * CC^−1^”. The sEPSC were automatically detected using the event detection package of axograph. This package uses a pre-established template (amplitude: 5 pA, rise-time: 0.6 ms, decay time: 3.5 ms, baseline: 1.5 ms, length: 4 ms) for detect synaptic events. Events with amplitude below 4 pA were rejected.

### Locomotor activity

Horizontal and vertical activities were monitored for 10 min at night (complete darkness) in an automated Omnitech Digiscan apparatus (AccuScan Instruments, Columbus, OH). The box had a dimension of 20 cm ×20 cm. Horizontal activities were detected by a set of infrared sensors ranged in XY axes while vertical activities were detected only in the X axis. The distance between each detector was 2.5 cm. Recordings and data processing were made using the software VersaMax 4.20 (Molecular Devices). Horizontal and vertical activities were expressed by the number of beam interruptions, split in 15-s intervals. For instance, we evaluated the number of beam interruptions for a few simple movements: 2–4 for a head movement (left to right), 4–5 for a 90-degree rotation and 3–6 for a 5-cm movement.

### Exploration/recognition test

The object recognition task is based on the spontaneous tendency of rodents to explore a novel object longer than a familiar one. We opted for a non-stressful cognitive test because 3xTg-AD mice display a high anxiety level [83], [115], which could confuse the interpretation of their behavioral response in more stressful cognitive tasks (such as the Morris water maze). During the conditioning phase, animals were placed individually in a standard mouse cage (29.2 cm ×19 cm ×12.7 cm) containing two objects for a period of 10 min. The objects were comparable in size, texture and shape complexity. The objects were either rectangular (4 cm ×4 cm ×6 cm), cylindrical (4 cm diameter ×6 cm high), conical (3.8 cm in diameter at the base and 1.8 cm in diameter in height, 4.5 cm high) or triangular (2.5 cm per side ×6.5 cm high). The test was repeated one hour later with a known object and a new one. Object recognition was investigated after one hour because the recognition index of our control mice was significantly decreased after this time, thus reducing the sensitivity of the test. The time spent exploring and sniffing each object was recorded. The ratio of time spent exploring the new versus the known object was used as an indicator of recognition and memory [116], [117], [118], [119]. Animals whose exploration was not considered sufficient to allow recognition (20 s per item) were rejected from the analysis.

### Physical and muscular evaluation

Animals were suspended by the forelimbs to a stretched cable of 2 mm diameter, and the time mice remained clinging to the cable was measured to assess muscular tone. Each animal underwent two tests and the best score was kept.

### Statistical Analysis

Values were expressed as mean ± standard error of the mean (SEM). Statistical comparisons were performed using a two-way ANOVA for the study of two variables simultaneously. When variable interaction was detected, statistical comparisons were performed using a one-way ANOVA followed by Tukey-Kramer post-hoc test. When only 2 groups were compared, statistical comparisons were performed using an unpaired Student's t-test. The DHA application protocol was analyzed using paired Student's t-test. Correlation analyses were performed using linear regressions. The relationship between molecular parameters (fatty acid content, synaptic and pathologic markers) and cellular electrophysiological parameters was calculated by attributing one electrophysiological value par animal (corresponding to the average of a minimum of two recording cells). Correlations between electrophysiological parameters were carried out using values from single neurons.

## Supporting Information

Figure S1 **Tissue preparation for electrophysiological recordings.** (A) Side-view of the mouse brain. The black line represents the 300-μm horizontal section used in this study. (B) Horizontal mouse brain section stained with haematoxylin nuclear counterstain. Whole-cell recordings (REC) were made in deep layer of EC. Abbreviations: CPu, caudate putamen (striatum); Hipp, hippocampus.
(TIFF)

Figure S2 **Cellular and membranous models to explain the modulation of CC on current density and voltage variation.** (A, B) The injected current is dispersed throughout the membrane surface. Thus, a neuron with a larger membrane surface area will have a smaller current density, since membrane conductance is kept constant. Consequently, this smaller current density produces a smaller voltage variation. Mathematical formulas to explain modulation of CC on resting potential at cellular and membranous level were given in panels C and D. Formula abbreviations: IR: input resistance (cellular resistance), CC: cell capacitance, G_cell_: cell conductance, Gm: membrane conductance, Iinj: injected current, Im: membrane current or current density, ΔV: voltage variation, ≈: proportional.
(TIF)

Table S1 Summary of the effects of DHA dietary treatment and transgene expression effects on proteins, animal weight and lipid content in the frontal cortex. Values for synaptic markers were normalized with actin. Values are expressed as mean ± SEM. Statistical comparisons were performed using a two-way ANOVA for the study of two variables simultaneously. When variable interaction was detected, statistical comparisons were performed using a one-way ANOVA followed by Tukey-Kramer post-hoc test. Abbreviations: AA, arachidonic acid; CAMKII: calcium/camodulin-dependent protein kinase II; DHA, docosahexaenoic acid; DS: detergent-soluble (membrane fraction); TBS: Tris buffer saline (cytosolic fraction); PAK: p21-activated kinase; PSD95: postsynaptic density-95. *P<0.05: **P<0.01 (significantly different of NonTg mice fed with control diet). ^o^P<0.05 (significantly different of NonTg mice fed with high-DHA diet). #P<0.05 (significantly different of 3xTg-AD mice fed with control diet).
(TIF)

Table S2 **Summary of DHA dietary treatment and transgene expression effects on pathologic markers in 3xTg-AD mice.** Values of tau in TBS-soluble fractions were expressed as a ratio over actin quantified on the same blots. TBS-soluble Aβ40 and Aβ42 values were expressed as picograms per milligram of protein while FAE-Aβ values were expressed as picograms per milligram of tissue. Tau in FAE fractions was quantified by relative optical density. Values are expressed as mean ± SEM and number of mice analysis is indicated between brackets. Statistical comparisons were performed using an unpaired Student's t-test. Abbreviations: TBS: Tris buffer saline; FAE: formic-acid extract (detergent-insoluble fraction). *P<0.05.
(TIF)

Table S3 **Summary of electrophysiology properties of deep layer pyramidal neurons from NonTg and 3xTg-AD mice fed with control or high-DHA diet.** Electrophysiologic data were obtained from 8 mice per groups. Values are expressed as mean ± SEM and number of recorded neurons is indicated between brackets. Statistical comparisons were performed using a two-way ANOVA and P value was given in the right column. When variable interaction was detected, statistical comparisons were performed using a one-way ANOVA followed by Tukey-Kramer posthoc test. ***P<0.001 (significantly different of other groups).
(TIF)

Table S4 **Summary of behavioral outcomes.** Values are expressed as mean ± SEM. Statistical comparisons were performed using a two-way ANOVA for the study of two variables simultaneously. When variable interaction was detected, statistical comparisons were performed using a one-way ANOVA followed by Tukey-Kramer post-hoc test.°P<0.05 (significantly different of 3xTg-AD mice fed with high-DHA diet).
(TIF)

Table S5 **Summary of the intrinsic properties of entorhinal cortex neurons before, during and after an application of 2 μM DHA.** Electrophysiologic data were obtained from 5 mice. Values are expressed as mean ± SEM. Statistical comparisons were performed using paired Student's t-test. Abbreviations: F-I, firing rate versus injected current.
(TIF)

Table S6 **Number of spontaneously active neurons in each group.** Electrophysiologic data were obtained from 8 mice per groups.
(TIF)

## References

[1] Duyckaerts C (2004). Looking for the link between plaques and tangles.. Neurobiol Aging.

[2] Counts SE, Nadeem M, Lad SP, Wuu J, Mufson EJ (2006). Differential expression of synaptic proteins in the frontal and temporal cortex of elderly subjects with mild cognitive impairment.. J Neuropathol Exp Neurol.

[3] Scheff SW, Price DA (2006). Alzheimer's disease-related alterations in synaptic density: neocortex and hippocampus.. J Alzheimers Dis.

[4] Palop JJ, Chin J, Mucke L (2006). A network dysfunction perspective on neurodegenerative diseases.. Nature.

[5] Palop JJ, Chin J, Roberson ED, Wang J, Thwin MT (2007). Aberrant excitatory neuronal activity and compensatory remodeling of inhibitory hippocampal circuits in mouse models of Alzheimer's disease.. Neuron.

[6] Braak H, Braak E (1991). Neuropathological stageing of Alzheimer-related changes.. Acta Neuropathol.

[7] Haroutunian V, Perl DP, Purohit DP, Marin D, Khan K (1998). Regional distribution of neuritic plaques in the nondemented elderly and subjects with very mild Alzheimer disease.. Arch Neurol.

[8] Haroutunian V, Purohit DP, Perl DP, Marin D, Khan K (1999). Neurofibrillary tangles in nondemented elderly subjects and mild Alzheimer disease.. Arch Neurol.

[9] Gomez-Isla T, Price JL, McKeel DW, Morris JC, Growdon JH (1996). Profound loss of layer II entorhinal cortex neurons occurs in very mild Alzheimer's disease.. J Neurosci.

[10] Dickerson BC, Salat DH, Greve DN, Chua EF, Rand-Giovannetti E (2005). Increased hippocampal activation in mild cognitive impairment compared to normal aging and AD.. Neurology.

[11] Scheff SW, Price DA, Schmitt FA, DeKosky ST, Mufson EJ (2007). Synaptic alterations in CA1 in mild Alzheimer disease and mild cognitive impairment.. Neurology.

[12] Terry RD, Masliah E, Salmon DP, Butters N, DeTeresa R (1991). Physical basis of cognitive alterations in Alzheimer's disease: synapse loss is the major correlate of cognitive impairment.. Ann Neurol.

[13] Dong H, Martin MV, Chambers S, Csernansky JG (2007). Spatial relationship between synapse loss and beta-amyloid deposition in Tg2576 mice.. J Comp Neurol.

[14] Jacobsen JS, Wu CC, Redwine JM, Comery TA, Arias R (2006). Early-onset behavioral and synaptic deficits in a mouse model of Alzheimer's disease.. Proc Natl Acad Sci U S A.

[15] Minkeviciene R, Rheims S, Dobszay MB, Zilberter M, Hartikainen J (2009). Amyloid beta-induced neuronal hyperexcitability triggers progressive epilepsy.. J Neurosci.

[16] Oddo S, Caccamo A, Kitazawa M, Tseng BP, LaFerla FM (2003). Amyloid deposition precedes tangle formation in a triple transgenic model of Alzheimer's disease.. Neurobiol Aging.

[17] Oddo S, Caccamo A, Shepherd JD, Murphy MP, Golde TE (2003). Triple-transgenic model of Alzheimer's disease with plaques and tangles: intracellular Abeta and synaptic dysfunction.. Neuron.

[18] Calon F, Cole G (2007). Neuroprotective action of omega-3 polyunsaturated fatty acids against neurodegenerative diseases: evidence from animal studies.. Prostaglandins Leukot Essent Fatty Acids.

[19] Boudrault C, Bazinet RP, Ma DW (2009). Experimental models and mechanisms underlying the protective effects of n-3 polyunsaturated fatty acids in Alzheimer's disease.. J Nutr Biochem.

[20] Calon F, Lim GP, Yang F, Morihara T, Teter B (2004). Docosahexaenoic acid protects from dendritic pathology in an Alzheimer's disease mouse model.. Neuron.

[21] Hooijmans CR, Van der Zee CE, Dederen PJ, Brouwer KM, Reijmer YD (2009). DHA and cholesterol containing diets influence Alzheimer-like pathology, cognition and cerebral vasculature in APPswe/PS1dE9 mice.. Neurobiol Dis.

[22] Green KN, Martinez-Coria H, Khashwji H, Hall EB, Yurko-Mauro KA (2007). Dietary docosahexaenoic acid and docosapentaenoic acid ameliorate amyloid-beta and tau pathology via a mechanism involving presenilin 1 levels.. J Neurosci.

[23] Lim GP, Calon F, Morihara T, Yang F, Teter B (2005). A diet enriched with the omega-3 fatty acid docosahexaenoic acid reduces amyloid burden in an aged Alzheimer mouse model.. J Neurosci.

[24] Oksman M, Iivonen H, Hogyes E, Amtul Z, Penke B (2006). Impact of different saturated fatty acid, polyunsaturated fatty acid and cholesterol containing diets on beta-amyloid accumulation in APP/PS1 transgenic mice.. Neurobiol Dis.

[25] Perez SE, Berg BM, Moore KA, He B, Counts SE (2010). DHA diet reduces AD pathology in young APPswe/PS1 Delta E9 transgenic mice: possible gender effects.. J Neurosci Res.

[26] Calon F, Lim GP, Morihara T, Yang F, Ubeda O (2005). Dietary n-3 polyunsaturated fatty acid depletion activates caspases and decreases NMDA receptors in the brain of a transgenic mouse model of Alzheimer's disease.. Eur J Neurosci.

[27] Yurko-Mauro K, McCarthy D, Rom D, Nelson EB, Ryan AS (2010). Beneficial effects of docosahexaenoic acid on cognition in age-related cognitive decline..

[28] Jones RS (1993). Entorhinal-hippocampal connections: a speculative view of their function.. Trends Neurosci.

[29] Sewards TV, Sewards MA (2003). Input and output stations of the entorhinal cortex: superficial vs. deep layers or lateral vs. medial divisions?. Brain Res Brain Res Rev.

[30] Woodhall GL, Bailey SJ, Thompson SE, Evans DI, Jones RS (2005). Fundamental differences in spontaneous synaptic inhibition between deep and superficial layers of the rat entorhinal cortex.. Hippocampus.

[31] Julien C, Tremblay C, Phivilay A, Berthiaume L, Emond V (2010). High-fat diet aggravates amyloid-beta and tau pathologies in the 3xTg-AD mouse model.. Neurobiol Aging.

[32] Bousquet M, Saint-Pierre M, Julien C, Salem N, Cicchetti F (2008). Beneficial effects of dietary omega-3 polyunsaturated fatty acid on toxin-induced neuronal degeneration in an animal model of Parkinson's disease.. FASEB J.

[33] Haag M (2003). Essential fatty acids and the brain.. Can J Psychiatry.

[34] Calder PC (2008). Polyunsaturated fatty acids, inflammatory processes and inflammatory bowel diseases.. Mol Nutr Food Res.

[35] Salem N (1989). Omega-3 fatty acids: molecular and biochemical aspects.. New protective roles for selected nutrients.

[36] Jarrard LE (1993). On the role of the hippocampus in learning and memory in the rat.. Behav Neural Biol.

[37] Izquierdo I, Medina JH (1997). Memory formation: the sequence of biochemical events in the hippocampus and its connection to activity in other brain structures.. Neurobiol Learn Mem.

[38] Lipton PA, Eichenbaum H (2008). Complementary roles of hippocampus and medial entorhinal cortex in episodic memory.. Neural Plast.

[39] Faumont S, Boulin T, Hobert O, Lockery SR (2006). Developmental regulation of whole cell capacitance and membrane current in identified interneurons in C. elegans.. J Neurophysiol.

[40] Gentet LJ, Stuart GJ, Clements JD (2000). Direct measurement of specific membrane capacitance in neurons.. Biophys J.

[41] Golowasch J, Thomas G, Taylor AL, Patel A, Pineda A (2009). Membrane capacitance measurements revisited: dependence of capacitance value on measurement method in nonisopotential neurons.. J Neurophysiol.

[42] Amatniek JC, Hauser WA, DelCastillo-Castaneda C, Jacobs DM, Marder K (2006). Incidence and predictors of seizures in patients with Alzheimer's disease.. Epilepsia.

[43] Rao SC, Dove G, Cascino GD, Petersen RC (2009). Recurrent seizures in patients with dementia: frequency, seizure types, and treatment outcome.. Epilepsy Behav.

[44] Sperling RA, Laviolette PS, O'Keefe K, O'Brien J, Rentz DM (2009). Amyloid deposition is associated with impaired default network function in older persons without dementia.. Neuron.

[45] Almeida CG, Tampellini D, Takahashi RH, Greengard P, Lin MT (2005). Beta-amyloid accumulation in APP mutant neurons reduces PSD-95 and GluR1 in synapses.. Neurobiol Dis.

[46] Sze CI, Troncoso JC, Kawas C, Mouton P, Price DL (1997). Loss of the presynaptic vesicle protein synaptophysin in hippocampus correlates with cognitive decline in Alzheimer disease.. J Neuropathol Exp Neurol.

[47] Robson LG, Dyall S, Sidloff D, Michael-Titus AT (2011). Omega-3 polyunsaturated fatty acids increase the neurite outgrowth of rat sensory neurones throughout development and in aged animals.. Neurobiol Aging.

[48] Wurtman RJ, Cansev M, Ulus IH (2009). Synapse formation is enhanced by oral administration of uridine and DHA, the circulating precursors of brain phosphatides.. J Nutr Health Aging.

[49] Sakamoto T, Cansev M, Wurtman RJ (2007). Oral supplementation with docosahexaenoic acid and uridine-5'-monophosphate increases dendritic spine density in adult gerbil hippocampus.. Brain Res.

[50] Cao D, Kevala K, Kim J, Moon HS, Jun SB (2009). Docosahexaenoic acid promotes hippocampal neuronal development and synaptic function.. J Neurochem.

[51] Hunt CA, Schenker LJ, Kennedy MB (1996). PSD-95 is associated with the postsynaptic density and not with the presynaptic membrane at forebrain synapses.. J Neurosci.

[52] Levinson JN, Chery N, Huang K, Wong TP, Gerrow K (2005). Neuroligins mediate excitatory and inhibitory synapse formation: involvement of PSD-95 and neurexin-1beta in neuroligin-induced synaptic specificity.. J Biol Chem.

[53] Prange O, Wong TP, Gerrow K, Wang YT, El-Husseini A (2004). A balance between excitatory and inhibitory synapses is controlled by PSD-95 and neuroligin.. Proc Natl Acad Sci U S A.

[54] King DL, Arendash GW (2002). Maintained synaptophysin immunoreactivity in Tg2576 transgenic mice during aging: correlations with cognitive impairment.. Brain Res.

[55] Martin RE, Wickham JQ, Om AS, Sanders J, Ceballos N (2000). Uptake and incorporation of docosahexaenoic acid (DHA) into neuronal cell body and neurite/nerve growth cone lipids: evidence of compartmental DHA metabolism in nerve growth factor-differentiated PC12 cells.. Neurochem Res.

[56] Hamilton JA, Guo W, Kamp F (2002). Mechanism of cellular uptake of long-chain fatty acids: Do we need cellular proteins?. Mol Cell Biochem.

[57] Chen CT, Liu Z, Ouellet M, Calon F, Bazinet RP (2009). Rapid beta-oxidation of eicosapentaenoic acid in mouse brain: an in situ study.. Prostaglandins Leukot Essent Fatty Acids.

[58] Scheff SW, Price DA, Schmitt FA, Mufson EJ (2006). Hippocampal synaptic loss in early Alzheimer's disease and mild cognitive impairment.. Neurobiol Aging.

[59] Ferrer I, Gullotta F (1990). Down's syndrome and Alzheimer's disease: dendritic spine counts in the hippocampus.. Acta Neuropathol (Berl).

[60] Robson LG, Dyall S, Sidloff D, Michael-Titus AT (2008). Omega-3 polyunsaturated fatty acids increase the neurite outgrowth of rat sensory neurones throughout development and in aged animals..

[61] Darios F, Davletov B (2006). Omega-3 and omega-6 fatty acids stimulate cell membrane expansion by acting on syntaxin 3.. Nature.

[62] Kamenetz F, Tomita T, Hsieh H, Seabrook G, Borchelt D (2003). APP processing and synaptic function.. Neuron.

[63] Busche MA, Eichhoff G, Adelsberger H, Abramowski D, Wiederhold KH (2008). Clusters of hyperactive neurons near amyloid plaques in a mouse model of Alzheimer's disease.. Science.

[64] van der Heyden MJ, Hilgevoord AA, Bour LJ, Ongerboer de Visser BW (1994). Modeling motoneuron firing properties: dependency on size and calcium dynamics.. Biol Cybern.

[65] Chang YM, Rosene DL, Killiany RJ, Mangiamele LA, Luebke JI (2005). Increased action potential firing rates of layer 2/3 pyramidal cells in the prefrontal cortex are significantly related to cognitive performance in aged monkeys.. Cereb Cortex.

[66] Sah P, Davies P (2000). Calcium-activated potassium currents in mammalian neurons.. Clin Exp Pharmacol Physiol.

[67] Sah P, Faber ES (2002). Channels underlying neuronal calcium-activated potassium currents.. Prog Neurobiol.

[68] Zhou FM, Hablitz JJ (1996). Layer I neurons of rat neocortex. I. Action potential and repetitive firing properties.. J Neurophysiol.

[69] Matthews EA, Weible AP, Shah S, Disterhoft JF (2008). The BK-mediated fAHP is modulated by learning a hippocampus-dependent task.. Proc Natl Acad Sci U S A.

[70] Lancaster B, Nicoll RA (1987). Properties of two calcium-activated hyperpolarizations in rat hippocampal neurones.. J Physiol.

[71] Xiao Y, Li X (1999). Polyunsaturated fatty acids modify mouse hippocampal neuronal excitability during excitotoxic or convulsant stimulation.. Brain Res.

[72] Young C, Gean PW, Chiou LC, Shen YZ (2000). Docosahexaenoic acid inhibits synaptic transmission and epileptiform activity in the rat hippocampus.. Synapse.

[73] Yuen AW, Sander JW, Fluegel D, Patsalos PN, Bell GS (2005). Omega-3 fatty acid supplementation in patients with chronic epilepsy: a randomized trial.. Epilepsy Behav.

[74] Voskuyl RA, Vreugdenhil M, Kang JX, Leaf A (1998). Anticonvulsant effect of polyunsaturated fatty acids in rats, using the cortical stimulation model.. Eur J Pharmacol.

[75] Taha AY, Burnham WM, Auvin S (2010). Polyunsaturated fatty acids and epilepsy..

[76] Hartley DM, Walsh DM, Ye CP, Diehl T, Vasquez S (1999). Protofibrillar intermediates of amyloid beta-protein induce acute electrophysiological changes and progressive neurotoxicity in cortical neurons.. J Neurosci.

[77] Sun XD, Mo ZL, Taylor BM, Epps DE (2003). A slowly formed transient conformer of Abeta(1-40) is toxic to inward channels of dissociated hippocampal and cortical neurons of rats.. Neurobiol Dis.

[78] Brunel N, Chance FS, Fourcaud N, Abbott LF (2001). Effects of synaptic noise and filtering on the frequency response of spiking neurons.. Phys Rev Lett.

[79] Kohn AF (1998). Effects of synaptic noise on a neuronal pool model with strong excitatory drive and recurrent inhibition.. Biosystems.

[80] Stacey WC, Lazarewicz MT, Litt B (2009). Synaptic noise and physiological coupling generate high-frequency oscillations in a hippocampal computational model.. J Neurophysiol.

[81] Prescott SA, De Koninck Y (2003). Gain control of firing rate by shunting inhibition: roles of synaptic noise and dendritic saturation.. Proc Natl Acad Sci U S A.

[82] Martinez-Coria H, Green KN, Billings LM, Kitazawa M, Albrecht M (2010). Memantine improves cognition and reduces Alzheimer's-like neuropathology in transgenic mice.. Am J Pathol.

[83] Clinton LK, Billings LM, Green KN, Caccamo A, Ngo J (2007). Age-dependent sexual dimorphism in cognition and stress response in the 3xTg-AD mice.. Neurobiol Dis.

[84] Chiu CC, Su KP, Cheng TC, Liu HC, Chang CJ (2008). The effects of omega-3 fatty acids monotherapy in Alzheimer's disease and mild cognitive impairment: a preliminary randomized double-blind placebo-controlled study.. Prog Neuropsychopharmacol Biol Psychiatry.

[85] Freund-Levi Y, Eriksdotter-Jonhagen M, Cederholm T, Basun H, Faxen-Irving G (2006). Omega-3 fatty acid treatment in 174 patients with mild to moderate Alzheimer disease: OmegAD study: a randomized double-blind trial.. Arch Neurol.

[86] Kotani S, Sakaguchi E, Warashina S, Matsukawa N, Ishikura Y (2006). Dietary supplementation of arachidonic and docosahexaenoic acids improves cognitive dysfunction.. Neurosci Res.

[87] Boston PF, Bennett A, Horrobin DF, Bennett CN (2004). Ethyl-EPA in Alzheimer's disease—a pilot study.. Prostaglandins Leukot Essent Fatty Acids.

[88] Fotuhi M, Mohassel P, Yaffe K (2009). Fish consumption, long-chain omega-3 fatty acids and risk of cognitive decline or Alzheimer disease: a complex association.. Nat Clin Pract Neurol.

[89] Quinn JF, Raman R, Thomas RG, Yurko-Mauro K, Nelson EB (2010). Docosahexaenoic acid supplementation and cognitive decline in Alzheimer disease: a randomized trial.. Jama.

[90] Morris MC, Evans DA, Bienias JL, Tangney CC, Bennett DA (2003). Consumption of fish and n-3 fatty acids and risk of incident Alzheimer disease.. Arch Neurol.

[91] Heude B, Ducimetiere P, Berr C (2003). Cognitive decline and fatty acid composition of erythrocyte membranes—The EVA Study.. Am J Clin Nutr.

[92] Huang TL, Zandi PP, Tucker KL, Fitzpatrick AL, Kuller LH (2005). Benefits of fatty fish on dementia risk are stronger for those without APOE epsilon4.. Neurology.

[93] Kroger E, Verreault R, Carmichael PH, Lindsay J, Julien P (2009). Omega-3 fatty acids and risk of dementia: the Canadian Study of Health and Aging.. Am J Clin Nutr.

[94] Devore EE, Grodstein F, van Rooij FJ, Hofman A, Rosner B (2009). Dietary intake of fish and omega-3 fatty acids in relation to long-term dementia risk.. Am J Clin Nutr.

[95] Whelan J, Rust C (2006). Innovative dietary sources of n-3 fatty acids.. Annu Rev Nutr.

[96] Kolanowski W, Laufenberg G, Kunz B (2004). Fish oil stabilisation by microencapsulation with modified cellulose.. Int J Food Sci Nutr.

[97] Hogan SA, O'Riordan ED, O'Sullivan M (2003). Microencapsulation and oxidative stability of spray-dried fish oil emulsions.. J Microencapsul.

[98] Mizushima Y, Harauchi T, Yoshizaki T, Makino S (1984). A rat mutant unable to synthesize vitamin C.. Experientia.

[99] Nejak-Bowen KN, Zeng G, Tan X, Cieply B, Monga SP (2009). Beta-catenin regulates vitamin C biosynthesis and cell survival in murine liver.. J Biol Chem.

[100] Mitchell JH, Cawood E, Kinniburgh D, Provan A, Collins AR (2001). Effect of a phytoestrogen food supplement on reproductive health in normal males.. Clin Sci (Lond).

[101] Wang H, Tranguch S, Xie H, Hanley G, Das SK (2005). Variation in commercial rodent diets induces disparate molecular and physiological changes in the mouse uterus.. Proc Natl Acad Sci U S A.

[102] Hsieh AT, Brenna JT (2009). Dietary docosahexaenoic acid but not arachidonic acid influences central nervous system fatty acid status in baboon neonates.. Prostaglandins Leukot Essent Fatty Acids.

[103] Brenna JT, Diau GY (2007). The influence of dietary docosahexaenoic acid and arachidonic acid on central nervous system polyunsaturated fatty acid composition.. Prostaglandins Leukot Essent Fatty Acids.

[104] Diau GY, Hsieh AT, Sarkadi-Nagy EA, Wijendran V, Nathanielsz PW (2005). The influence of long chain polyunsaturate supplementation on docosahexaenoic acid and arachidonic acid in baboon neonate central nervous system.. BMC Med.

[105] Dullemeijer C, Zock PL, Coronel R, Den Ruijter HM, Katan MB (2008). Differences in fatty acid composition between cerebral brain lobes in juvenile pigs after fish oil feeding.. Br J Nutr.

[106] Folch J, Lees M, Sloane Stanley GH (1957). A simple method for the isolation and purification of total lipides from animal tissues.. J Biol Chem.

[107] Masood A, Stark KD, Salem N (2005). A simplified and efficient method for the analysis of fatty acid methyl esters suitable for large clinical studies.. J Lipid Res.

[108] Arsenault D, Zhang ZW (2006). Developmental remodelling of the lemniscal synapse in the ventral basal thalamus of the mouse.. J Physiol.

[109] Zhang ZW, Arsenault D (2005). Gain modulation by serotonin in pyramidal neurones of the rat prefrontal cortex.. J Physiol.

[110] Thid D, Benkoski JJ, Svedhem S, Kasemo B, Gold J (2007). DHA-induced changes of supported lipid membrane morphology.. Langmuir.

[111] Serth J, Lautwein A, Frech M, Wittinghofer A, Pingoud A (1991). The inhibition of the GTPase activating protein-Ha-ras interaction by acidic lipids is due to physical association of the C-terminal domain of the GTPase activating protein with micellar structures.. Embo J.

[112] Namani T, Ishikawa T, Morigaki K, Walde P (2007). Vesicles from docosahexaenoic acid.. Colloids Surf B Biointerfaces.

[113] Bruno MJ, Koeppe RE, Andersen OS (2007). Docosahexaenoic acid alters bilayer elastic properties.. Proc Natl Acad Sci U S A.

[114] Hamilton JA, Brunaldi K (2007). A model for fatty acid transport into the brain.. J Mol Neurosci.

[115] Sterniczuk R, Antle MC, Laferla FM, Dyck RH (2010). Characterization of the 3xTg-AD mouse model of Alzheimer's disease: part 2. Behavioral and cognitive changes.. Brain Res.

[116] Balderas I, Rodriguez-Ortiz CJ, Salgado-Tonda P, Chavez-Hurtado J, McGaugh JL (2008). The consolidation of object and context recognition memory involve different regions of the temporal lobe.. Learn Mem.

[117] Winters BD, Saksida LM, Bussey TJ (2008). Object recognition memory: neurobiological mechanisms of encoding, consolidation and retrieval.. Neurosci Biobehav Rev.

[118] de Lima MN, Luft T, Roesler R, Schroder N (2006). Temporary inactivation reveals an essential role of the dorsal hippocampus in consolidation of object recognition memory.. Neurosci Lett.

[119] Akirav I, Maroun M (2006). Ventromedial prefrontal cortex is obligatory for consolidation and reconsolidation of object recognition memory.. Cereb Cortex.

