# Supplementary figures and images for: DHA Improves Cognition and Prevents Dysfunction of Entorhinal Cortex Neurons in 3xTg-AD Mice
Source: PLoS One. 2011 Feb 23;6(2):e17397. doi: 10.1371/journal.pone.0017397 (PMC3044176; doi:10.1371/journal.pone.0017397)

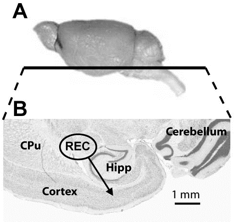

Supplement: Figure S1 — Tissue preparation for electrophysiological recordings. (A) Side-view of the mouse brain. The black line represents the 300-μm horizontal section used in this study. (B) Horizontal mouse brain section stained with haematoxylin nuclear counterstain. Whole-cell recordings (REC) were made in deep layer of EC. Abbreviations: CPu, caudate putamen (striatum); Hipp, hippocampus. (TIFF) [file pone.0017397.s001.tiff]

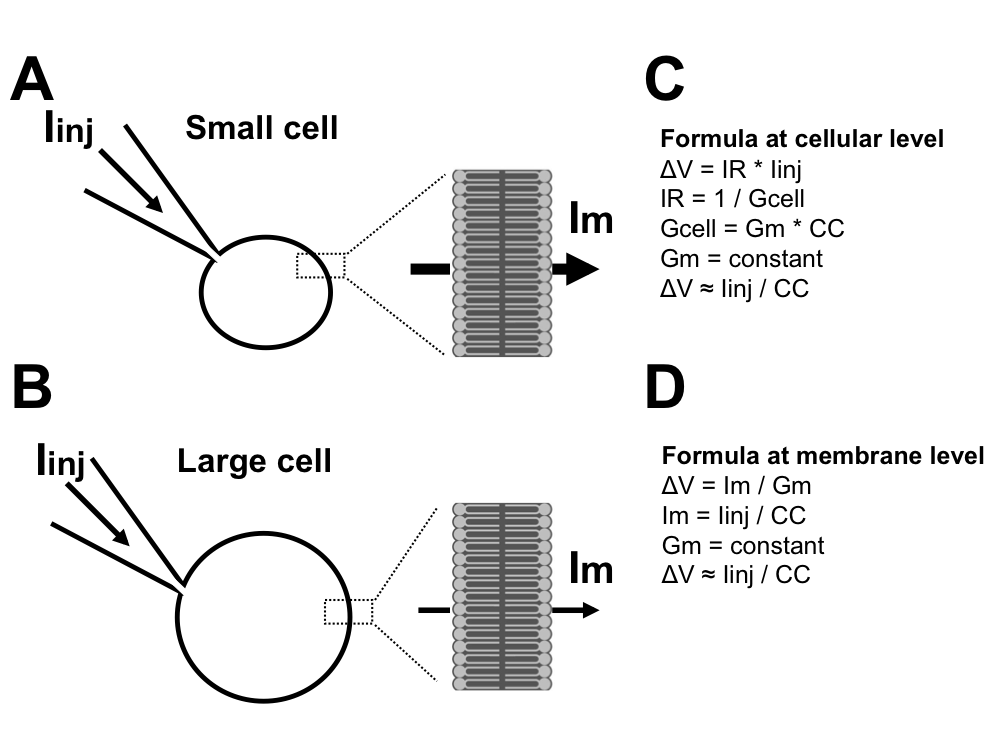

Supplement: Figure S2 — Cellular and membranous models to explain the modulation of CC on current density and voltage variation. (A, B) The injected current is dispersed throughout the membrane surface. Thus, a neuron with a larger membrane surface area will have a smaller current density, since membrane conductance is kept constant. Consequently, this smaller current density produces a smaller voltage variation. Mathematical formulas to explain modulation of CC on resting potential at cellular and membranous level were given in panels C and D. Formula abbreviations: IR: input resistance (cellular resistance), CC: cell capacitance, Gcell: cell conductance, Gm: membrane conductance, Iinj: injected current, Im: membrane current or current density, ΔV: voltage variation, ≈: proportional. (TIF) [file pone.0017397.s002.tif]

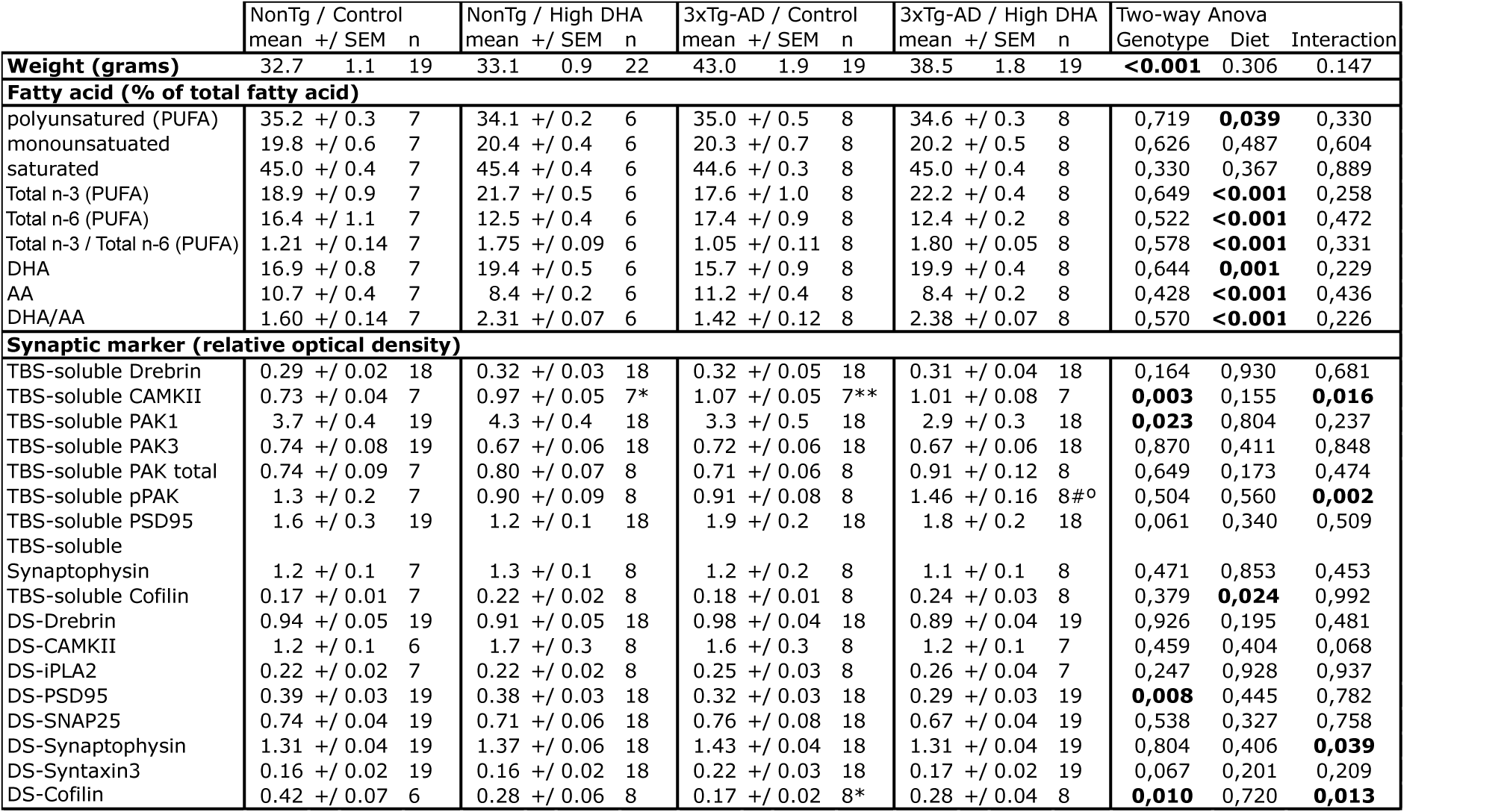

Supplement: Table S1 — Summary of the effects of DHA dietary treatment and transgene expression effects on proteins, animal weight and lipid content in the frontal cortex. Values for synaptic markers were normalized with actin. Values are expressed as mean ± SEM. Statistical comparisons were performed using a two-way ANOVA for the study of two variables simultaneously. When variable interaction was detected, statistical comparisons were performed using a one-way ANOVA followed by Tukey-Kramer post-hoc test. Abbreviations: AA, arachidonic acid; CAMKII: calcium/camodulin-dependent protein kinase II; DHA, docosahexaenoic acid; DS: detergent-soluble (membrane fraction); TBS: Tris buffer saline (cytosolic fraction); PAK: p21-activated kinase; PSD95: postsynaptic density-95. *P<0.05: **P<0.01 (significantly different of NonTg mice fed with control diet). oP<0.05 (significantly different of NonTg mice fed with high-DHA diet). #P<0.05 (significantly different of 3xTg-AD mice fed with control diet). (TIF) [file pone.0017397.s003.tif]

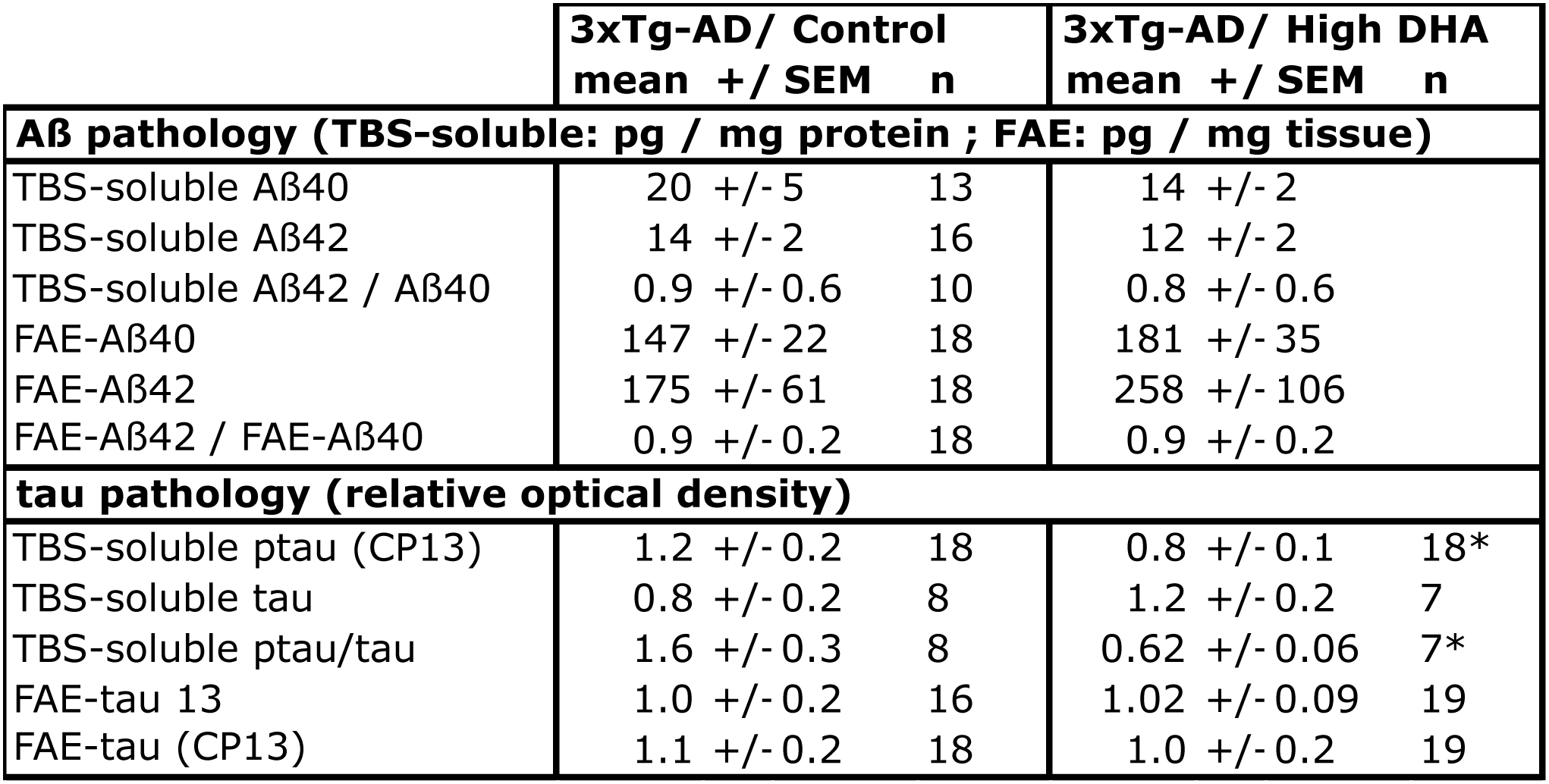

Supplement: Table S2 — Summary of DHA dietary treatment and transgene expression effects on pathologic markers in 3xTg-AD mice. Values of tau in TBS-soluble fractions were expressed as a ratio over actin quantified on the same blots. TBS-soluble Aβ40 and Aβ42 values were expressed as picograms per milligram of protein while FAE-Aβ values were expressed as picograms per milligram of tissue. Tau in FAE fractions was quantified by relative optical density. Values are expressed as mean ± SEM and number of mice analysis is indicated between brackets. Statistical comparisons were performed using an unpaired Student's t-test. Abbreviations: TBS: Tris buffer saline; FAE: formic-acid extract (detergent-insoluble fraction). *P<0.05. (TIF) [file pone.0017397.s004.tif]

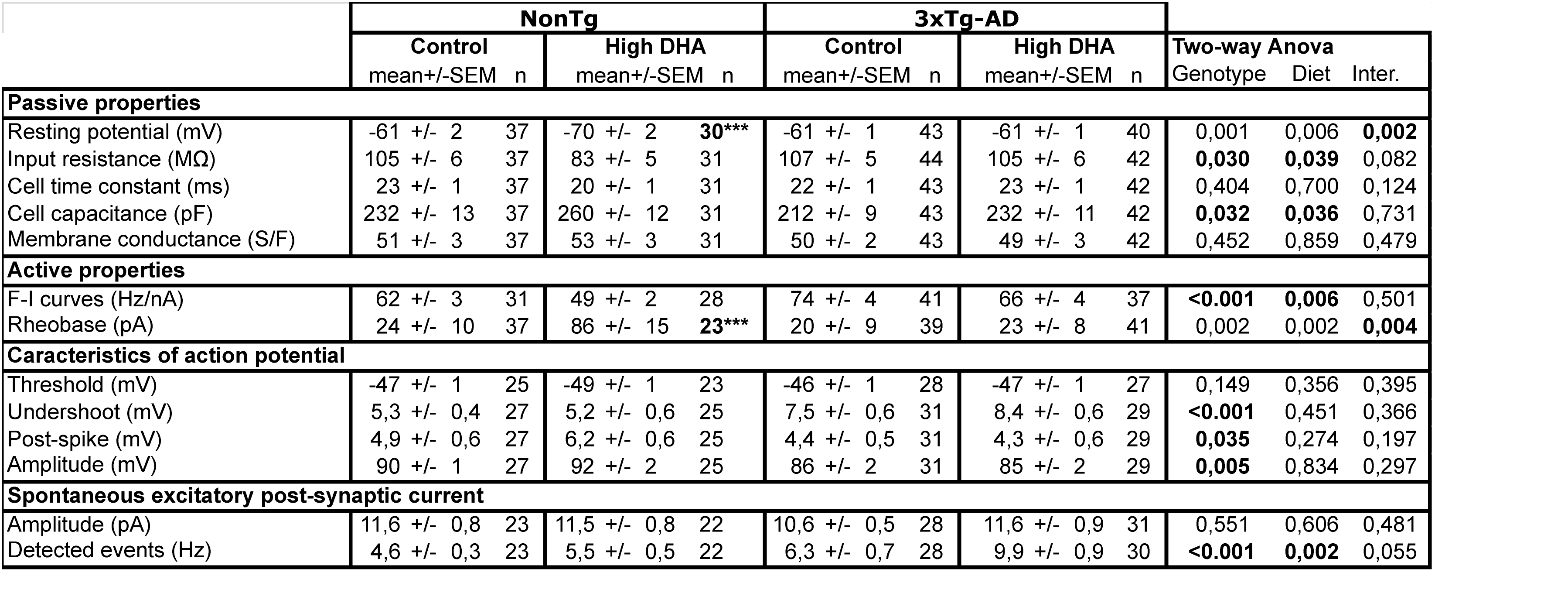

Supplement: Table S3 — Summary of electrophysiology properties of deep layer pyramidal neurons from NonTg and 3xTg-AD mice fed with control or high-DHA diet. Electrophysiologic data were obtained from 8 mice per groups. Values are expressed as mean ± SEM and number of recorded neurons is indicated between brackets. Statistical comparisons were performed using a two-way ANOVA and P value was given in the right column. When variable interaction was detected, statistical comparisons were performed using a one-way ANOVA followed by Tukey-Kramer posthoc test. ***P<0.001 (significantly different of other groups). (TIF) [file pone.0017397.s005.tif]

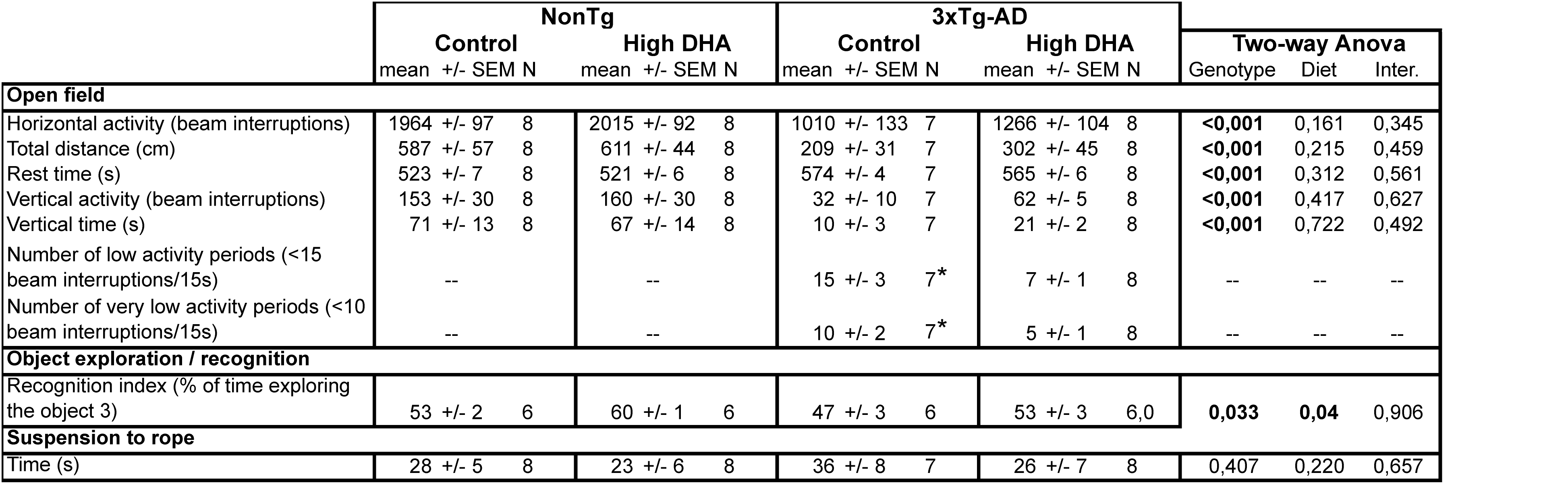

Supplement: Table S4 — Summary of behavioral outcomes. Values are expressed as mean ± SEM. Statistical comparisons were performed using a two-way ANOVA for the study of two variables simultaneously. When variable interaction was detected, statistical comparisons were performed using a one-way ANOVA followed by Tukey-Kramer post-hoc test.°P<0.05 (significantly different of 3xTg-AD mice fed with high-DHA diet). (TIF) [file pone.0017397.s006.tif]

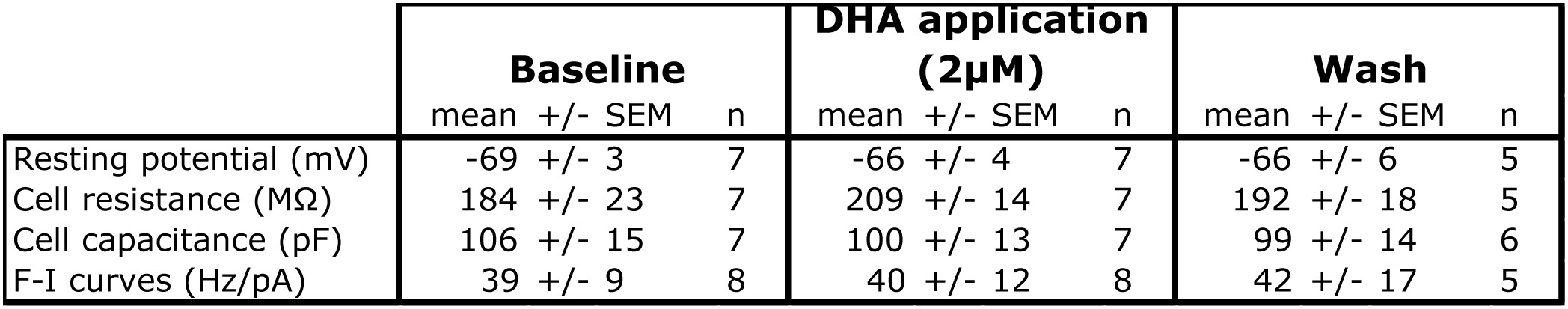

Supplement: Table S5 — Summary of the intrinsic properties of entorhinal cortex neurons before, during and after an application of 2 μM DHA. Electrophysiologic data were obtained from 5 mice. Values are expressed as mean ± SEM. Statistical comparisons were performed using paired Student's t-test. Abbreviations: F-I, firing rate versus injected current. (TIF) [file pone.0017397.s007.tif]

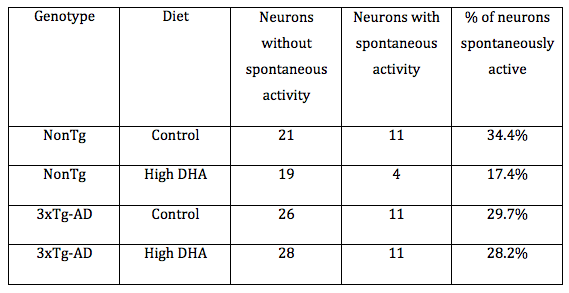

Supplement: Table S6 — Number of spontaneously active neurons in each group. Electrophysiologic data were obtained from 8 mice per groups. (TIF) [file pone.0017397.s008.tif]
